# Genetic associations with learning over 100 days of practice

**DOI:** 10.1038/s41539-022-00121-2

**Published:** 2022-05-04

**Authors:** Cherry Youn, Andrew D. Grotzinger, Christina M. Lill, Lars Bertram, Florian Schmiedek, Martin Lövdén, Ulman Lindenberger, Michel Nivard, K. Paige Harden, Elliot M. Tucker-Drob

**Affiliations:** 1 Department of Psychology, University of Texas at Austin, Austin, TX USA; 2 Lübeck Interdisciplinary Platform for Genome Analytics, University of Lübeck, Lübeck, Germany; 3 Aging Epidemiology Unit, School of Public Health, Imperial College London, London, UK; 4 Center for Lifespan Changes in Brain and Cognition, Department of Psychology, University of Oslo, Oslo, Norway; 5 Department for Education and Human Development, DIPF|Leibniz Institute for Research and Information in Education, Frankfurt am Main, Germany; 6 Center for Lifespan Psychology, Max Planck Institute for Human Development, Berlin, Germany; 7 Aging Research Center, Karolinska Institutet, Stockholm, Sweden; 8 Department of Psychology, University of Gothenburg, Gothenburg, Sweden; 9 Max Planck UCL Centre for Computational Psychiatry and Ageing Research, Berlin, Germany, and London UK; 10 Department of Biological Psychology, Vrije Universiteit Amsterdam, Amsterdam, The Netherlands; 11 Population Research Center, University of Texas at Austin, Austin, TX USA

**Keywords:** Human behaviour, Human behaviour

## Abstract

Cognitive performance is both heritable and sensitive to environmental inputs and sustained practice over time. However, it is currently unclear how genetic effects on cognitive performance change over the course of learning. We examine how polygenic scores (PGS) created from genome-wide association studies of educational attainment and cognitive performance are related to improvements in performance across nine cognitive tests (measuring perceptual speed, working memory, and episodic memory) administered to 131 adults (*N* = 51, ages = 20–31, and *N* = 80, ages = 65–80 years) repeatedly across 100 days. We observe that PGS associations with performance on a given task can change over the course of learning, with the specific pattern of change in associations differing across tasks. PGS correlations with pre-test to post-test scores may mask variability in how soon learning occurs over the course of practice. The associations between PGS and learning do not appear to simply reconstitute patterns of association between baseline performance and subsequent learning. Associations involving PGSs, however, were small with large confidence intervals. Intensive longitudinal research such as that described here may be of substantial value for clarifying the genetics of learning when implemented as far larger scale.

## Introduction

Intelligence, educational attainment (EA), and academic performance are all substantially heritable, meaning that much of the variation in outcomes is attributable to genetic differences^1–7^. Intelligence has often been conceptualized as the ability to learn^8^, and has also been shown to be sensitive to learning. EA and academic performance are themselves conceptualized as outcomes of prolonged learning that has occurred, and may themselves be predictive of future, ongoing, learning. Thus, all three phenotypes implicitly involve a dynamic process of learning that unfolds over time. Here, we provide one of the first direct tests that examines how static differences in DNA sequence are associated with the dynamic process of learning. Theoretically, the concept of the “reaction norm” describes how genetic variation is more appropriately thought of as governing a dynamic phenotypic response to environmental input, rather than as governing a fixed end state^9–11^. Yet theoretical reaction norm conceptions have rarely been tested vis-à-vis complex human phenotypes like cognition.

**Fig. 1:**
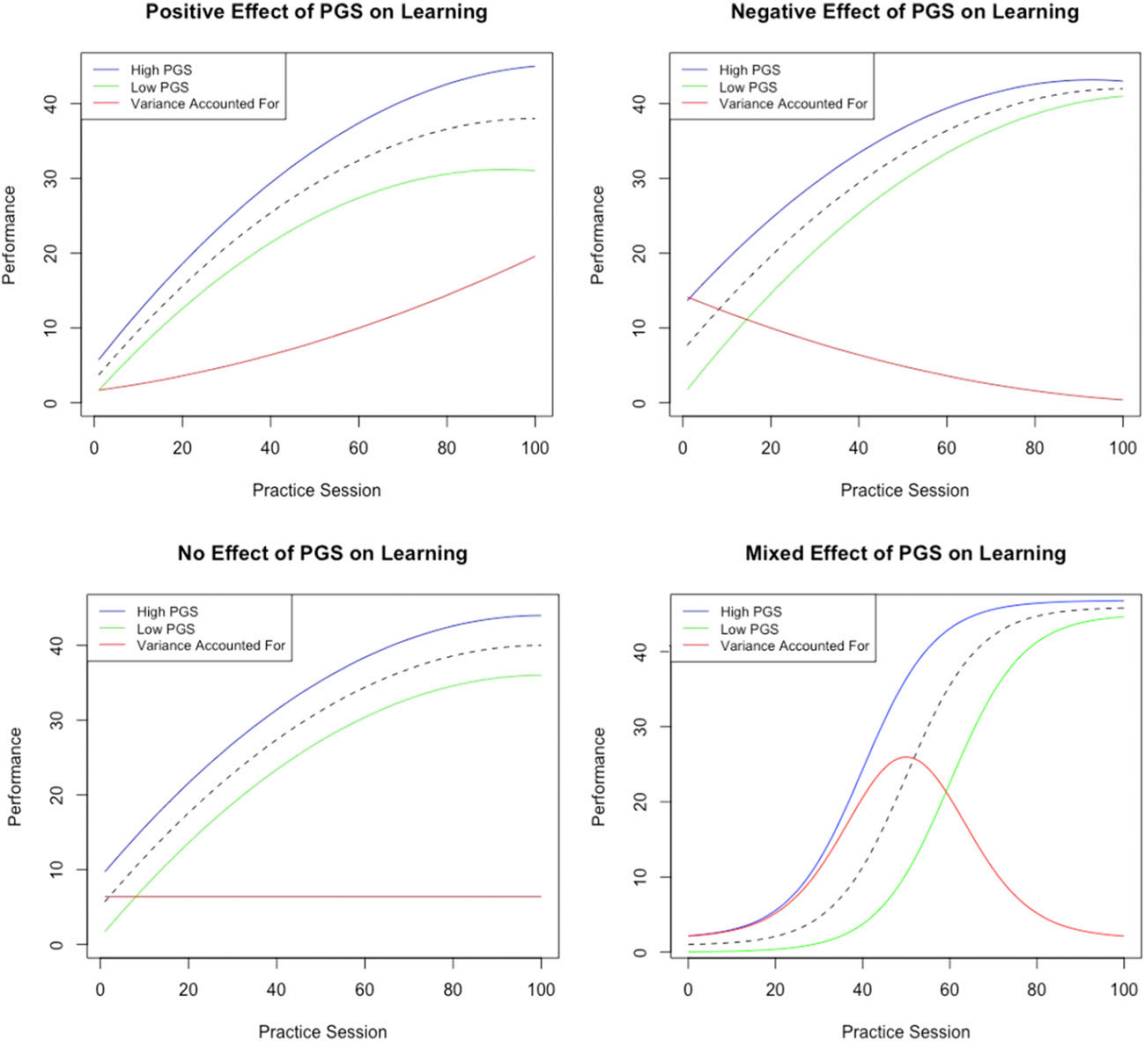
Conceptualization of the genetic effects on rates of learning. Top left figure is an example of magnification of genetic effects on initial levels of performance across time, where there are differences in the slope of learning between high and low PGS groups and the differences translate to more variance accounted for by genes. Top right figure is an example of a negative association between PGS and rates of change, where the genetic effects on initial levels of performance decrease over time and the experience equalizes differences between the two groups. Bottom left figure is an example that shows no association between the PGS and rates of change, indicating that the genetic effects remain constant across the environmental range and there is no genetic effect of PGS on learning. Lastly, bottom right figure is an example that shows a combination of all three patterns.

In the context of cognitive and academic learning, genetic effects are often assumed to be consistently amplified over the course of a learning regime^12^. However, other potential patterns are possible (Fig. 1). For instance, exposure to practice may reduce the effect of genetic differences in cognitive performance in a compensatory pattern: practice gives individuals at lower levels of initial performance additional opportunity to increase their skill while producing relatively less benefit to those who are already performing at closer to maximum performance levels. Alternatively, exposure to practice may benefit all individuals uniformly, thereby increasing mean levels of performance without changing the magnitude of differences in performance across genotypes. A combination of all three patterns is also possible: during early phases of practice, genetic effects on performance might be magnified, as some individuals learn faster than others. Later, however, those who were initially slower to learn eventually make large gains with practice, while those who have already gained remain at close to asymptote. Cross-over interactions, where genotypes related to the highest ultimate skill acquisition exhibit the lowest initial levels of performance during early phases of practice, are also possible but perhaps least likely.

Moreover, how differences in genotype relate to the shape and rate of skill acquisition might be universal across cognitive tasks, or it may differ by content domain or superficial demands of the to-be-learned task^13^. Outside of a genetic context, Ackerman reported that tasks requiring speed and accuracy of motor movements often exhibit decreased inter-individual variability with practice^14^, particularly when several alternative approaches or strategies can be developed to perform the tasks. In such scenarios, content ability, like verbal and math skill, may play a role during early phases of skill acquisition during which time individuals have identified the task demands and determined an efficient strategy, but become less relevant during later phases of skill acquisition^15,16^.

In contrast, when learning more complex tasks that rely less on motor movement and speed, Ackerman reported that whether individuals diverge vs. converge over the course of practice is primarily determined by whether there is a bound on the upper range of performance^14,17^. Tasks in which the upper reasonable range of performance is bounded are known as “closed” tasks, and performance typically converges with practice (e.g., a “catch up” pattern). Conversely, for “open” tasks in which the upper range of performance is unbounded, “rich-get-richer” effects are typically observed, where individual differences in intelligence, working memory, and performance are magnified with practice. As genetically informative research on learning across different cognitive tasks is rare, whether such patterns are observed with respect to convergence and divergence of heritable variation in task performance is an open question. We emphasize the necessity of detecting different patterns of genetic effects on the shape of learning because effects such as “catch up” and “rich-get-richer” may pose new questions in research as well as challenges for educational systems.

Few studies have used longitudinal data to estimate the role of genetics in individual differences in rate of learning over time. One exception is a previous twin study by Fox et al.^18^, which concluded that the heritability of performance increased across multi-trial motor skill learning. Studies by Hambrick and Tucker-Drob and by Mosing et al. found that music practice was not only substantially heritable^19,20^, but also genetically associated with musical expertize and accomplishment. These results suggest a genetic effect on rate of skill acquisition. Here, we supplement previous research by examining genetic associations with rate of learning using a polygenic score (PGS) approach and estimating learning on nine cognitive tasks spanning three cognitive domains over 100 days of intensive practice.

To examine genetic correlates of learning, we use a PGS derived from the genome-wide association studies (GWAS) of EA (*N* = 1,131,881) and cognitive performance (CP; *N* = 257,841)^4,21^. These GWAS of EA and CP have reported that the corresponding EAPGS and CPPGS explain 9% and up to 4% of the variance in cognitive outcomes, respectively^4,21^, although prediction may of course vary by sample characteristics due to issues of both portability and statistical variation.

We examine how these same PGS relate to rate and shape of learning on nine tasks that measure episodic memory, working memory, and perceptual speed across 100 days of practice. During practice phase, the difficulties of all tasks, except for the three comparison tasks, were individualized based on pre-test performance. This individualization allows for the experimental deconfounding of individual differences in rate and shape of learning. In other words, by tailoring task difficulty to individual pre-test scores, individuals did not differ as greatly from one another in their distance from an upper reasonable bound on performance. Furthermore, adaptive task difficulty challenges even the high performing participants to ensure that all sessions are utilized as effective learning opportunities. Finally, we examined PGS prediction among some of the most extreme scores within the sample, building off recent observations that extreme PGS can be particularly predictive^22^. Owing to the intensive nature of the protocol, with extensive pre- and post-testing, and 100 days of practice on nine different cognitive tasks (for a total of 155,002 observations), the sample size (*N* = 131) is relatively small by the standards of genetic research. As such, our study is exploratory, emphasizing the conceptual models of change in individual differences and descriptive patterns of results rather than null hypothesis significance tests.

## Results

### Most people improve over time, but people differ in their rate of learning

Supplementary Fig. 3 presents a ridge plot of the distribution of the standardized differences between pre- and post-test scores, classified by task types. Standardized post-test scores represent the difference between the pre- and post-test and the standard deviation of the difference in pre-test units. All mean differences for each of the nine cognitive tasks are positive, suggesting that on average, performance improved during the practice phase and most individuals scored higher at post-test than at pre-test (Supplementary Table 3). Furthermore, substantial standard deviations that range from 0.75 to over 1 indicate considerable individual differences in learning. The 95%-ile range of changes is largely positive, but also includes some people who decreased in performance at post-test compared to pre-test.

### Genetic associations with overall learning from the beginning and end of practice are modest and unreliable

#### Regression models of individual tasks

The simplest way to model genetic associations with learning is to estimate the associations of PGSs with task performance at the beginning and the end of practice, and with the overall change in performance over the course of practice. In our first set of analyses, we fitted linear regression models to test the associations between (1) EAPGS and pre-test scores, (2) EAPGS and post-test scores, and (3) EAPGS and the difference between pre- and post-test scores (Table 1). In line with the power analysis, we conducted one-tailed tests of statistical significance and standardized pre- and post-test scores relative to the mean and standard deviation of the pre-test scores. EAPGS was positively correlated with pre-test scores of N-Back Spatial and pre- and post-test scores of Number-Noun. In contrast, EAPGS was negatively correlated with pre-test scores (mean correct response times) of Figural/Spatial Comparison, indicating that individuals with higher EAPGS tend to respond slower. This may be explained by the response time-accuracy tradeoff, where participants with higher EAPGS prioritize higher accuracy at the expense of slower speed. EAPGS was also negatively correlated with the difference between pre-test and post-test scores on the N-Back. This suggests that the EAPGS negatively affects the rate of learning for this task and practice equalizes performance: individuals with lower EAPGS “catch up” to individuals with higher EAPGS by the end of the practice phase. We also conducted the same analyses with CPPGS and EA, which are detailed in the Supplementary Information (see Supplementary Tables 4–6).

**Table 1:** Linear regression analysis between EAPGS and test scores by cognitive task.

	Pre-test scores		Post-test scores		Difference	
	*Estimate*	*SE*	*Estimate*	*SE*	*Estimate*	*SE*
Episodic Memory Tasks						
Word List Memory Task	0.074	0.095	0.050	0.062	0.042	0.094
Number-Noun Pairs	0.189^a^	0.091	0.134^a^	0.098	0.086	0.124
Object Position Memory	0.093	0.094	−0.000	0.082	−0.121	0.107
Working Memory Tasks						
Alpha Span	−0.030	0.104	0.010	0.087	0.092	0.150
Memory Updating Numerical	0.039	0.108	0.063	0.117	0.026	0.141
N-Back Spatial	0.159^a^	0.092	−0.004	0.088	−0.245^a^	0.112
Perceptual Speed Tasks						
Numerical Comparison	−0.065	0.088	−0.023	0.027	−0.019	0.029
Verbal Comparison	−0.049	0.088	−0.037	0.042	−0.047	0.057
Figural/Spatial Comparison	−0.199^a^	0.086	−0.046	0.031	−0.034	0.039

*EAPGS* educational attainment polygenic score, *SE* standard error.

^a^Regression is significant at the 0.05 level (one-tailed).

#### Latent difference score models

Many of the results from the regression analyses show small effects that are very close to 0 and failed to reject the null after Bonferroni correction for multiple testing (*p* < 0.05). Observed difference scores for individual variables may be problematic due to the influence of measurement error, which can reduce power and bias associations toward zero^23,24^. We therefore went on to conduct analyses at the level of latent factors representing the individual ability domains, utilizing latent difference score modeling (LDSM) in M*plus*, version 8 (see Supplementary Notes and Supplementary Fig. 4)^13,25,26^, to represent error free change in the latent factors. This has the added benefit of increasing power to detect correlates of changes that occur at the level of the broad ability domains, rather than for the individual tasks. For LDSM analyses, we used the full sample, including non-genotyped individuals via maximum-likelihood estimation, for the measurement models to increase the stability in the estimation of the cognitive structure. Measurement invariance across time was imposed to ensure that the latent difference score could be meaningfully interpreted. To account for the possible role of age^22,27–29^, we included age group and PGS as covariates along with the interactions between PGS and age group (i.e., EA/CPPGS × age; see Supplementary Tables 7–12 for parameter estimates). Analyses produced similar patterns to those described earlier: despite grouping cognitive tasks by type to aggregate power across related outcomes, all but one pathway from PGS to the pre- or the latent difference score were small in magnitude and statistically nonsignificant. The interactions with age were also nonsignificant, indicating that stronger effects were not masked by the aggregation of data from younger and older participants. In contrast, we found consistently significant pathways (1) from age to the pre-test factor model and (2) from age to the latent difference score.

### People may differ in the shape of learning over 100 days

**Fig. 2:**
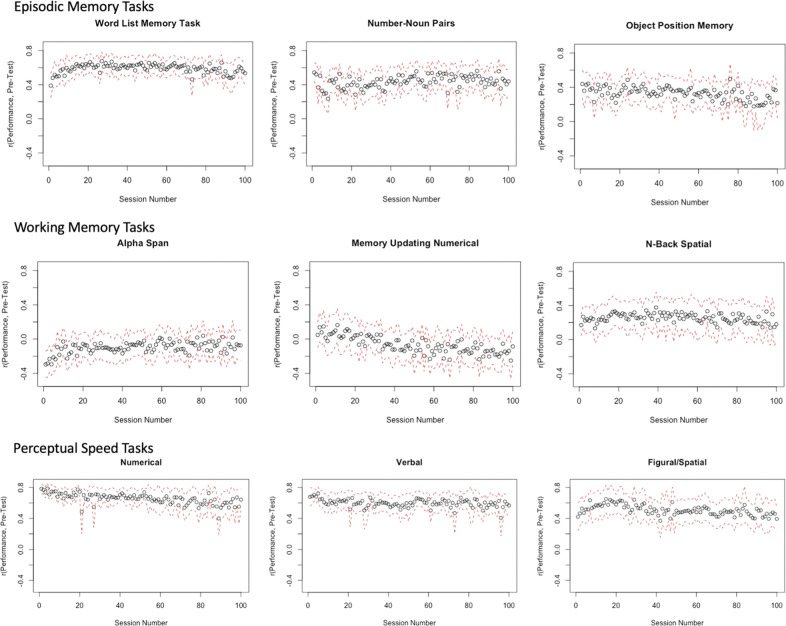
Paneled plot of correlations between pre-test scores and performance on all cognitive tasks over the practice phase. The black circles indicate correlations between pre-test scores and performance, and the red dotted lines indicate 95% confidence intervals.

Examining only the overall difference in task performance from the beginning to the end of practice might mask heterogeneity in the shape of learning over that time. To probe for potential differences in the shape of learning, we correlated pre-test scores with performance at every practice session for each task (Fig. 2). Correlations between pre-test scores and the average performance across blocks of 10 days and by age group are in Supplementary Figs. 5 and 6, respectively. Correlations in the first practice sessions for all perceptual speed tasks ranged from 0.40 to 0.80, indicating moderate to strong differences between participants with high and low scores at baseline. On the other hand, correlations in the first practice sessions for all episodic memory tasks and the N-Back ranged from ~0.20 to 0.50, indicating modest differences between participants with high and low scores at baseline. In contrast, the average correlations in the first practice sessions for Alpha Span and Memory Updating were −0.20 and 0, respectively, indicating little to no differences between participants with high and low scores at baseline.

Although most results were nonsignificant, we qualitatively observed three stylized patterns of change in the correlations between pretest scores and practice session performance across the sessions: upward trend, downward trend, and constant. The upward trend of correlations is seen most prominently in Alpha Span, indicating that higher pretest scores were associated with both greater initial performance and greater learning across time. In contrast, the downward trends in correlations across practice sessions is seen for Object Position, Memory Updating, and Numerical Comparison. This pattern indicates that participants with high pre-test scores start practicing with slight advantage, but the difference between high and low score groups equalize over time, and even lead to the low score group performing better for Memory Updating.

All other tasks show correlations that generally remain constant throughout the practice phase. This trend suggests that participants with high scorers begin with slight advantage, and its effects remain constant throughout the practice phase. However, note that we were only able to qualitatively describe these patterns, and cannot make strong statistical inferences given the large confidence intervals, and nonsignificant nature, of most parameter estimates.

### Genetics of performance over the 100 days of learning

**Fig. 3:**
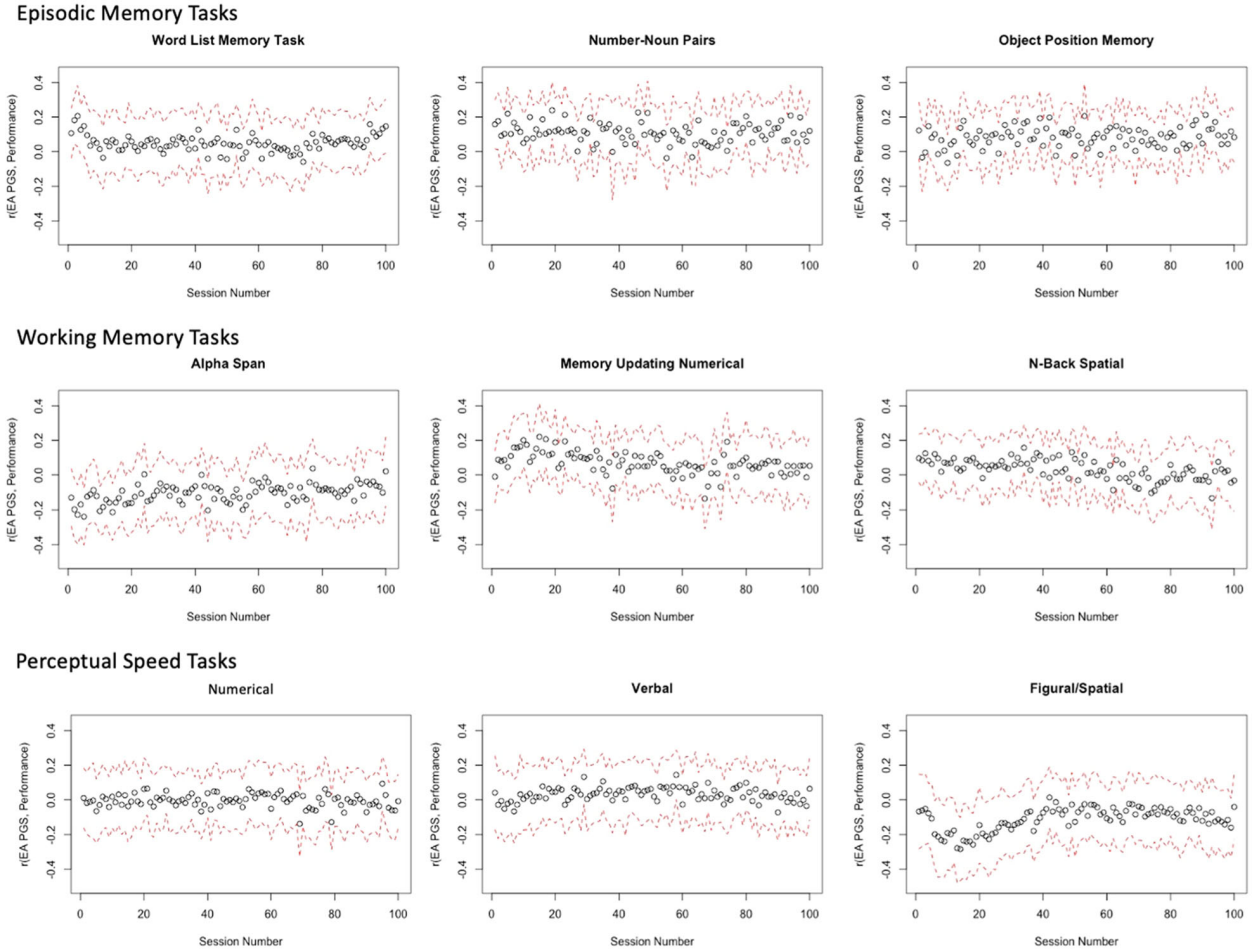
Paneled plot of correlations between EAPGS and performance on all cognitive tasks over the practice phase. The black circles indicate correlations between EAPGS and performance, and the red dotted lines indicate 95% confidence intervals.

We examined performance over the practice phase across every day of practice (Fig. 3), across blocks of 10 days (Supplementary Fig. 7), and by age group (Supplementary Fig. 8) as correlates of EAPGS to examine how genetic associations transform with learning. Pre-test scores, PGS, and cognitive scores at all individual waves were computed to be independent of age at pre-test and PC of ancestry before analyses to calculate their residuals. With residuals of variables that were independent of baseline age and PC, three different trajectories of correlations were qualitatively observed: upward trend, downward trend, and constant. Correlations for Alpha Span showed an upward trend from ~−0.25 to 0.05, indicating that participants with lower EAPGS perform better at baseline, but the difference between the high and low EAPGS equalize toward the end of practice. In contrast, Memory Updating and N-Back showed a downward trend in correlations to ~*r* = 0, such that initial EAPGS-associated differences in performance decreased with practice.

All other tasks show correlations that generally remain constant throughout the practice phase. The correlations and their trajectories remain similar, even when creating means for blocks of ten sessions (Supplementary Fig. 7). As the interpretation of between-person differences in the daily practice data is complicated by the individualized presentation times, we also looked at the PGS correlations within groups who share the same presentation time and confirmed that the different patterns were not artifacts produced by the individualization procedure.

We also examined performance as correlates of CPPGS (see Supplementary Figs. 9–11). Like that of EAPGS, we identified different patterns of trajectories that do not appear to simply reconstitute patterns of association between baseline performance and subsequent learning. Details about pre-test scores as correlates of subsequent performance and performance as correlates of CPPGS are included in the Supplementary Information.

**Fig. 4:**
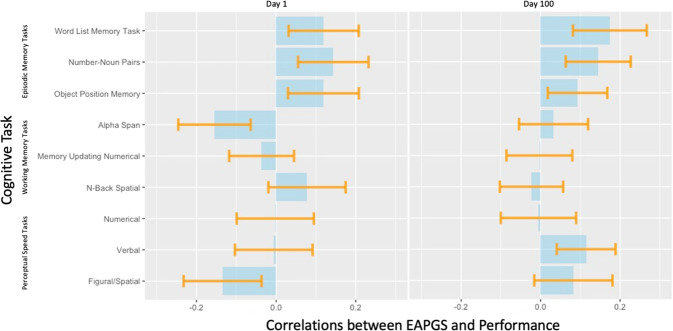
Spearman correlations between EAPGS and performance at day 1 (top) and at day 100 of the practice period (bottom) on all cognitive tasks. The blue bars indicate Spearman correlation coefficients between EAPGS and test performance and orange lines indicate error bars (i.e., ± standard error). Spearman correlations were used to reduce the potential influence of outliers.

Figure 4 includes correlations between EAPGS and performance at day 1 and EAPGS and performance at day 100 of the practice phase. Spearman correlations between CPPGS and performance at day 1 and 100 of the practice phase are also shown in Supplementary Fig. 12. To assess the standard errors of the correlation coefficients, we performed the bootstrapping method with 1,000 bootstraps for each cognitive task. We used the Spearman correlations to reduce the potential influence of outliers. Correlations between EAPGS and performance at day 1 show little to no differences, with correlations only ranging up to 0.155. However, more than half of the cognitive tasks (Word List, Alpha Span, Memory Updating, Verbal Comparison, Figural/Spatial Comparison) show an increase in correlations between EAPGS and performance at day 100. A similar trend is observed from correlations between CPPGS and initial performance at day 1 to correlations between CPPGS and performance at day 100, where the same five cognitive tasks showed an increase in correlations over the practice phase.

### Growth models of individual differences in change in cognitive performance

We formally modelled individual trajectories of learning by fitting linear, logarithmic, and exponential growth models to observations of cognitive performance in every task (Supplementary Fig. 13). We incorporated EAPGS into the models and examined correlates of learning by including it as a predictor of all parameters for which there were random effects. Fit statistics and key parameter estimates, including AIC of model comparisons, for exponential and logarithmic models are presented in Supplementary Table 13. Results indicated substantial variability in the shape and rate of learning. However, there were no statistically significant associations between the EAPGS and this variation. This may be attributable to lack of statistical power, as it is currently unknown what size the effect of genetic variation on rate and shape of learning may be. Thus, in the next section, we use the parameter estimates, regardless of significance, to produce expectations for trajectories at extreme PGSs, which we compare to observed trajectories of groups with extreme PGSs.

### Patterns of learning in individuals with extreme PGS

**Fig. 5:**
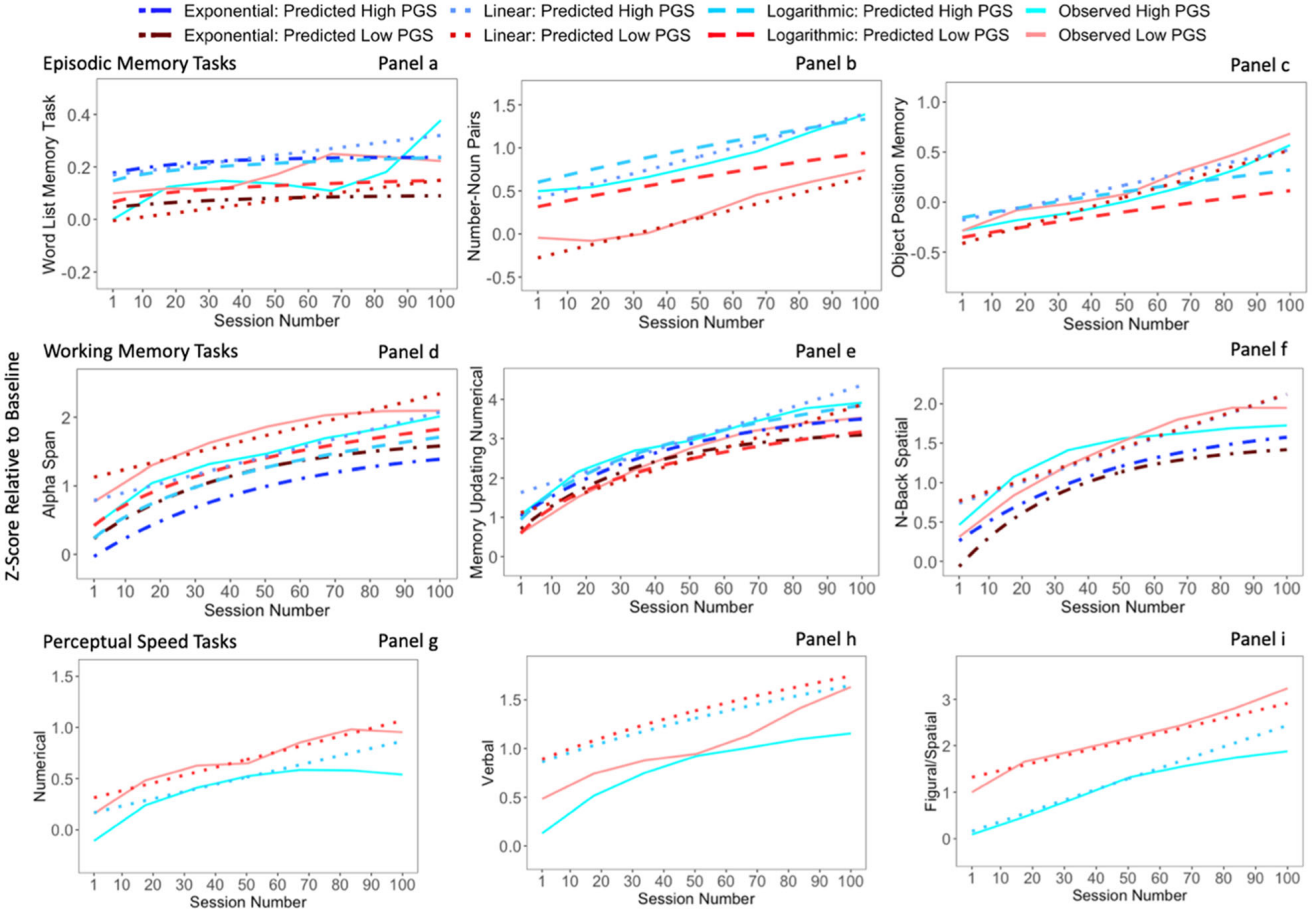
Paneled plot of means in performance over the practice phase. Subgroup means are created by extreme PGS groups (i.e., 15 participants with the lowest EAPGS vs. 15 participants with the highest EAPGS) on all cognitive tasks.

We examined how the rate and shape of learning differed between individuals with the most extreme PGS values (Fig. 5)— the highest 15 and lowest 15 EAPGS values in the samples. The two groups have a relatively equal balance of older and younger participants in each group, where the low EAPGS group (mean *z*-score = −0.9059; range = −2.0808–−0.2635) includes six older participants and nine younger participants, and the high EAPGS group (mean *z*-score = 0.9059; range = 0.164–1.4496) consists of eight older and seven younger participants. All outcomes are scaled relative to the observed mean and standard deviation at day 1 to ensure that all *z*-scores are relative to the distributions of initial task performance. We considered exponential and logarithmic functions, but they were not included in the figure if there was no convergence.

Descriptive results are plotted in Fig. 5. The predicted means in performance for all tasks except for Word List (Panel a) and N-Back (Panel f) follow relatively parallel trajectories for high and low PGS groups. Parallel trajectories indicate that differences in performance across the two groups remains over the course of the practice, such that genetic effects remain constant across the duration of practice.

Descriptive results for Word List (Panel a) indicate that although the high and low groups share similar average performance, trajectories of performance across practice appear to differ. The low PGS group starts the practice phase with an advantage, but the average performance remains relatively constant from the first to last session, with the group even showing a slight downward trend in performance starting at around session 60. In contrast, high PGS group shows a steeper rate of learning, with average performances dramatically increasing both at the beginning and the end of the practice phase.

Descriptive results for N-Back (Panel f) indicate that low and high PGS groups start at very similar points, with the high PGS group at a slight performance advantage relative to the low PGS group, and the low PGS group learning more over practice and ultimately performing better than high PGS group.

## Discussion

We investigated how PGS for EA and cognitive function relate to intensive learning over 100 days of practice. By examining PGS associations across the full learning period, rather than simply before and after learning provided the opportunity to observe the patterns by which correlations transformed over the course of learning. Despite the intensive longitudinal nature of the study design, the sample size did not appear to be sufficiently powered to detect PGS associations at statistically significant levels and we found no evidence that the correlation between performance and the PGS changes significantly over the course of learning. However, we observed a variety of qualitative patterns of change in PGS associations with cognitive performance over the course of the learning period.

One qualitative pattern was characterized by decreasing PGS-performance correlations with practice, indicating gradual equalization of performance over the course of learning, where participants with low PGS “catch up” to those with high PGS over the practice phase. A second qualitative pattern was marked by relatively constant correlations over the course of practice, such that individuals learn relatively equivalently across the range of PGS.

The qualitative patterns that we observed were inconsistent across tasks, even within the same ability domain. Moreover, the observed associations between PGS and learning do not appear to simply reconstitute patterns of association between baseline performance and subsequent learning. Rather, each cognitive task appeared to show a unique pattern of PGS associations with performance over practice, and patterns did not appear to cluster by the type of cognitive ability that was being measured, even in extreme PGS groups. Despite common speculations that genetic correlates of task performance will magnify over the course of learning, we did not observe this trend generally across the tasks.

It is important to call attention to several limitations. First, results involving the PGSs were imprecise and did not appear to be well powered. Investigations with larger sample sizes are required to yield more precise estimates of genetic correlates of learning. Such studies are costly to conduct, as a tremendous amount of time is required from each participant. Not only was the sample size small, but the PGS underperformed in terms of effect on EA in this specific sample (*R*^2^ = 1.97% [0.01%, 9.75%]) when compared with reasonable expectations based on previous associations with EA^4^. Importantly, this considerable underperformance PGS prediction of EA at 2% undermines our expectations regarding what might be reasonably observed for PGS prediction of learning over time. Indeed, in light of this underperformance in polygenic prediction of EA, it appears that our study was underpowered to predict variation in learning. Second, this sample was composed of individuals of European ancestry in Germany. Whether the current findings generalize to other populations is an open question, and we should consider this work exploratory given low power and absence of pre-registration. Lastly, it is unclear whether the observed gains in performance should be interpreted as of practice-related gains in performance on cognitive abilities unique to the individual tasks or as general gains in the general cognitive abilities shared across tasks. Although some evidence for transfer of training in the COGITO study has been previously reported^30^, other studies have generally failed to find that training or practice on specific cognitive tasks transfers more generally to other external tasks or to real-life skills^31^. Importantly, there is strong evidence that the long-term, intensive, multimodal forms of learning characteristic of formal education have general effects beyond the specific material taught^32,33^. Notwithstanding these considerations, we believe that understanding the mechanisms of task-specific learning may still provide important insights into the processes that govern learning more generally.

We investigated how EA/CPPGS relate to improvements in learning across 100 days and found that there does not appear to be a strong correlation between PGS and any cognitive measures. Although estimates were imprecise, our exploratory study is original in design and relevant for future studies that examine the role of genetic in individual differences. The qualitative patterns observed in the current study suggest that PGS associations with performance may change over the course of learning, with the pattern of change varying across tasks. Although it is commonly hypothesized that pre-existing differences in performance will be amplified by practice, it is also possible to observe “catch-up effects”, whereby performance differences narrow over the course of practice or training. Future research on the associations between genetics and learning will benefit from considering that changes between pre- and post-test scores may mask changes that occur over the course of learning that reflects when the learning occurs, and that genetic associations with learning may not simply reconstitute patterns of association between baseline performance and subsequent learning.

## Method

### Participants

This study included 51 younger (age range: 20–31 years, *M* = 25.6, SD = 2.7) and 80 older adults (age range: 65–80 years, *M* = 70.4, SD = 4.0) from the COGITO study, a study of variability in cognitive performance over 100 days of measurement^34^. All research conducted by the COGITO study was approved by the Max Planck Institute for Human Development, Berlin and adhered to all ethical regulations regarding human subjects. The COGITO study included a total sample of 101 younger and 103 older adults with a wide range of EA (range: 3–26 years, *M* = 15.0, SD = 3.8), but our analyses are restricted to participants for whom genetic data were available. All participants provided written informed consent to take part in the study.

### PGS

PGS were computed based on GWAS summary data from recent large-scale GWAS of EA and CP^4^. As participants from the COGITO study also participated in the Berlin Aging Study II (BASE-II)^35^, and due to restrictions by the 23andMe corporation on the sharing of summary GWAS data, summary data were obtained for a version of the Lee et al.’s GWAS that excluded BASE-II and 23andMe^4^. Genome-wide SNP genotyping in BASE-II was performed using the “Genome-Wide Human SNP Array 6.0” from Affymetrix Inc. and then processed as previously described (REF PMID 26821332). We constructed EA/CPPGS using a 500 kb clumping window, a pruning *R*^2^ threshold of 0.25, and a *p* value threshold of 1.0 (all SNPs). We did not consider other *p* value thresholds because we did not have a separate tuning sample available for multi-trial learning that would be required for such an analysis. EA/CPPGS were standardized within our sample (*M* = 0, SD = 1) and adjusted for the top 10 principal components (PC). This process identified no outliers in terms of ancestry (see Supplementary Fig. 1 for a scatterplot matrix of the PC). The PCs were estimated in a reference sample with European ancestry (1000 Genomes Project, phase 1) and subsequently projected on to BASE-II participants using the EIGENSTRAT software^36^. Previous work in the Twins Early Development Study have reported that the EAPGS based on summary statistics that exclude 23andMe data may account for upwards of 10% of the variance in some cognitive and achievement test scores^37,38^, although it is possible that this estimate is higher than would be obtained in other samples. Moreover, as our focal analysis did not examine static levels of cognition or achievement, but examined change in cognitive performance over time, it is unclear what a reasonable expectation for *R*^2^ should be. It is possible that by focusing specifically on change over time, pre-existing sources of variation are controlled, such that effect sizes are larger than are typically obtained for static levels. Alternatively, it is possible that learning in a discrete period of time produces relatively little reordering of individuals, such that effect sizes are smaller than are typically obtained for static levels that themselves represent aggregation of learning across many years. We therefore consider power across a range of *R*^2^ values. A power analysis for *R*^2^ ranging from <1 to 10% is reported in Supplementary Fig. 2. For an *R*^2^ of 9%, power is 97% to detect an association in this sample at alpha of 5%. However, for an *R*^2^ of 2%, power is <50%.

### Procedure

The COGITO study protocol consisted of three primary phases: an extensive pre-test evaluation, a practice phase of ~100 days (*M* = 101) on 12 cognitive tasks, and a post-test evaluation in which the same measures administered during pre-test were re-administered. Three choice reaction time tasks were excluded from our study, leaving with nine cognitive tasks for analyses. The practice phase included daily sessions that consisted of twelve different cognitive tasks. The average time elapsed between pre- and post-test was 197 days for the younger group, and 188 for the older group.

### Measures

Cognitive tasks were selected to measure episodic memory, working memory, and processing speed. Tasks were selected such that they could be used in a repeated fashion over multiple occasion.

#### Pre- and post-tests

Participants underwent 10 days of pre-testing with baseline measures of cognitive abilities. Based on pre-test performance, each participant was given different masking time, presentation time, or interstimulus interval at the practice phase. All tasks were tailored to each participant except for processing speed tasks. For all other tasks, difficulty levels at the practice phase were individualized by using different presentation time based on the average accuracies achieved at four different presentation time levels pre-test^34^. After the practice phase, participants completed post-testing. The same test blocks presented at pre-test were repeated at post-test, but without practice blocks.

Pre- and post-test scores for episodic and working memory tasks were calculated by averaging the proportion of correct trials across four different presentation times. As perceptual speed tasks were not individualized based on pre-test performance, no mean response time was calculated. Instead, we calculated the reciprocal of the mean correct response time from correct response time to calculate pre- and post-test scores. In doing so, higher scores translate to better performance across all tasks. The use of only correct responses is preceded by publications on the COGITO study.

#### Cognitive tasks

All cognitive tasks were selected to measure three fundamental aspects of commonly measured cognitive abilities, including episodic memory, working memory, and perceptual speed. The visual, numerical, and figural/spatial versions of the episodic memory tasks included: Word List, Number-Noun, and Object Position, respectively. The verbal, numerical, and spatial versions of the working memory are adapted versions of the Alpha Span^39^, Memory Updating^40^, and N-Back^41^, respectively. These tasks have shown to be valid indicators to test working memory^42^. Lastly, perceptual speed was measured with numerical, verbal, and figural/spatial versions of comparison tasks. More detailed descriptions of each cognitive task are reported in Supplementary Table 1.

### Analytic approach

Specifications for all growth models are found in Supplementary Table 2. We used the NLMIXED function within SAS to fit nonlinear growth curve models of changes in cognitive performance task in 131 participants over 100 days of practice. The NLMIXED procedure can be found on OSF: https://mfr.osf.io/render?url=https%3A%2F%2Fosf.io%2Fn2h3k%2Fdownload. We considered exponential and logarithmic models, each of which can capture learning responses consistently increasing at early stages but have diminishing marginal rates, as is typical for learning^43^. We fit exponential and logarithmic models to the observations (i.e., correlates between EAPGS and performance) and compared the correlates of learning between two groups that are distinguished by EAPGS.

### Data preprocessing

To remove the possible collinearity of the analytic variables with age and ancestry, we computed residuals of EA/CPPGS using linear regression models that predicted each PGS, and cognitive scores at pre-test, post-test, and practice phase from age and PCs of ancestry. Age was used as a continuous variable to run partial correlations and maintain consistency with ancestry variables. Results were similar when age was used as a categorical variable.

### Reporting summary

Further information on research design is available in the Nature Research Reporting Summary linked to this article.

## Supplementary information

Supplementary Information

Reporting Summary

## Data Availability

The data that support the findings of this study are available on request from the corresponding authors [F.S., M.L., & U.L.] from the COGITO study. The data are not publicly available as the collection, storage, use, and disclosure of personal data that could compromise research participant privacy or consent are strictly regulated in Germany. Data are however available from the authors upon reasonable request and with permission of the COGITO study. Supplementary information is available at *npj Science of Learning*’s website.
