# Supplementary material for: Genetic associations with learning over 100 days of practice
Source: NPJ Sci Learn. 2022 May 4;7:7. doi: 10.1038/s41539-022-00121-2 (PMC9068685; doi:10.1038/s41539-022-00121-2)
Supplement: Supplementary file 1 — Supplementary Information [file 41539_2022_121_MOESM1_ESM.pdf]

Supplementary Information

Genetic Associations with Learning Over 100 Days of Practice

Cherry Youn[1], Andrew D. Grotzinger[1], Christina M. Lill[2,3], Lars Bertram[2,4], Florian Schmiedek[5,6], Martin Lövdén[7,8], Ulman Lindenberger[6,9], Michel Nivard[10], K. Paige Harden[1,11], Elliot M. Tucker-Drob[1,11]

[1]Department of Psychology, University of Texas at Austin, Austin, TX

[2]Lübeck Interdisciplinary Platform for Genome Analytics, University of Lübeck, Lübeck, Germany

[3]Aging Epidemiology Unit, School of Public Health, Imperial College London, London, United Kingdom

[4]Center for Lifespan Changes in Brain and Cognition, Department of Psychology, University of Oslo, Oslo, Norway

[5]Department for Education and Human Development, DIPF | Leibniz Institute for Research and Information in Education, Frankfurt am Main, Germany

[6]Center for Lifespan Psychology, Max Planck Institute for Human Development, Berlin, Germany

[7]Aging Research Center, Karolinska Institutet, Stockholm, Sweden

[8]Department of Psychology, University of Gothenburg, Gothenburg, Sweden

[9]Max Planck UCL Centre for Computational Psychiatry and Ageing Research, Berlin, Germany, and London, United Kingdom

[10]Department of Biological Psychology, Vrije Universiteit Amsterdam, The Netherlands

[11]Population Research Center, University of Texas at Austin, Austin, TX

Correspondence to:

Cherry Youn, Department of Psychology, The University of Texas at Austin, 108 E. Dean Keeton Street, Stop A8000, Austin, TX 78712, USA. E-mail: youncherry@utexas.edu

KEYWORDS: polygenic scores, genetics, educational attainment, cognitive performance, learning

1. Supplementary Notes *4*

a. Linear regression analysis between CPPGS and test scores by cognitive task **4**

b. Linear regression analysis between educational attainment and test scores by cognitive task ... **4**

c. Genetic association with learning from pre-test to post-test using LDSM **4**

d. Performance as correlates of pre-test scores by age group **5**

e. Performance as correlates of EAPGS by age group **5**

f. Performance as correlates of CPPGS **6**

2. Supplementary Figure Legends *7*

Supplementary Figure 1. **Scatterplot matrix of the 10 principal components (PC)** **7**

Supplementary Figure 2. **Estimated power of the model with respect to the predictive power of PGS.** **8**

Supplementary Figure 3. **Ridge plot of the distribution of the standardized differences between pre-test and post-test scores by task type.** **9**

Supplementary Figure 4. **Path diagram of the LDSM used in the analyses.** **10**

Supplementary Figure 5. **Paneled plot of correlations between pre-test scores and performance on all cognitive tasks over the training phase across blocks of 10 days.** **11**

Supplementary Figure 6. **Paneled plot of correlations between pre-test scores and performance on all cognitive tasks over the training phase by age group.** **12**

Supplementary Figure 7. **Paneled plot of correlations between EAPGS and performance on all cognitive tasks over the training phase across blocks of 10 days.** **13**

Supplementary Figure 8. **Paneled plot of correlations between EAPGS and performance on all cognitive tasks over the training phase by age group.** **14**

Supplementary Figure 9. **Paneled plot of correlations between CPPGS and performance on all cognitive tasks over the training phase.** **15**

Supplementary Figure 10. **Paneled plot of correlations between CPPGS and performance on all cognitive tasks over the training phase across blocks of 10 days** **16**

Supplementary Figure 11. **Paneled plot of correlations between CPPGS and performance on all cognitive tasks over the training phase by age group.** **17**

Supplementary Figure 12. **Spearman correlations between CPPGS and performance at day 1 (top) and at day 100 of the training period (bottom) on all cognitive tasks.** **18**

Supplementary Figure 13. **Paneled plot of means in performance over the training phase for all participants on all cognitive tasks** **19**

3. Supplementary Tables *20*

Supplementary Table 1. **Descriptions of Cognitive Tasks** **20**

Supplementary Table 2. **Growth model equations** **22**

Supplementary Table 3. **Difference between pre-test scores and post-test scores** **24**

Supplementary Table 4. **Linear regression analysis between CPPGS and test scores by cognitive task** **25**

Supplementary Table 5. **Linear regression analysis between educational attainment and test scores by cognitive task....26**

Supplementary Table 6. **RMSEA and CFI for the LDSM for each PGS and task type....27**

Supplementary Table 7. **Parameter estimates from standardized age × EAPGS latent difference score models for episodic memory tasks....28**

Supplementary Table 8. **Parameter estimates from standardized age × EAPGS latent difference score models for working memory tasks....29**

Supplementary Table 9. **Parameter estimates from standardized age × EAPGS latent difference score models for processing speed tasks....30**

Supplementary Table 10. **Parameter estimates from standardized age × CPPGS latent difference score models for episodic memory tasks....31**

Supplementary Table 11. **Parameter estimates from standardized age × CPPGS latent difference score models for working memory tasks....32**

Supplementary Table 12. **Parameter estimates from standardized age × CPPGS latent difference score models for processing speed tasks....33**

Supplementary Table 13. **Model fit comparisons and key parameter estimates for regression models....34**

4. Supplementary References....36

1. Supplementary Notes

a. Linear regression analysis between CPPGS and test scores by cognitive task. We fitted linear regression models to test whether the associations between (1) CPPGS and pre-test scores, (2) CPPGS and post-test scores, and (3) CPPGS and the difference between pre-test and post-test scores were unique of one another. Results are presented in Supplementary Table 4. Pre- and post-test scores were transformed based on the pre-test mean and standard deviation by cognitive task and EAPGS were standardized for analyses. CPPGS displayed nonsignificant associations with all pre- and post-test scores. However, CPPGS was positively correlated with the difference between pre-test and post-test scores on the Word List Memory Task. This suggests that the CPPGS positively affects the rate of learning for this task over 100 days of practice.

b. Linear regression analysis between educational attainment and test scores by cognitive task. We fitted linear regression models to observe the influence of education on learning between (1) years of educational attainment and pre-test scores, (2) years of educational attainment and post-test scores, and (3) years of educational attainment and the difference between pre-test and post-test scores. To do this, pre- and post-test scores were transformed based on the pre-test mean and standard deviation by cognitive task and years of educational attainment was standardized for analyses. Results are presented in Supplementary Table 5. Years of educational attainment displayed significant associations with pre-test scores for all cognitive tasks. Similarly, we observed significant positive associations between educational attainment and post-test scores for all cognitive tasks except for Numerical Comparison. Word List Memory Task, Number-Noun Pairs, Verbal Comparison, and Figural/Spatial Comparison showed significant associations between years of educational attainment and the difference between pre- and post-test scores.

c. Genetic association with learning from pre-test to post-test using LDSM. Using LDSM, we modeled change for all latent cognitive variables and examined how age, PGS, or the interaction of age and PGS predicted pre-test performance and the difference in performance between pre- and post-test. The schematic representation of the LDSM is shown in Supplementary Figure 4. In the model, we included three latent factors that measure the construct. A latent factor that measures the construct is specified for each of two time points, pre-test (pre) and post-test (post). The third latent variable, delta (Δ), is a latent difference variable constructed to measure the latent change in the construct between pre and post-test. Observed models include age, PGS, interaction of age and PGS, and pre- and post-test of cognitive tasks. As both EAPGS and CPPGS were included in separate LDSM by task type, we estimated a total of six models using Mplus, version 8[1].

The model fits were evaluated using the Root Mean Square Error of Approximation (RMSEA) and Confirmatory Fit Index (CFI) with cut-off criteria of 0.06 and 0.95 for fit indexes based on previous proposal, respectively[2]. See Supplementary Table 6 for RMSEA and CFI for all models. The RMSEA was below this criterion for all models except for the two processing speed models. As lower RMSEA indicates better fit, the results from the processing speed models should be interpreted with caution. Similarly, the CFI was above the 0.95 criterion for all models except for the processing speed models, indicating good fit for the other models.

Using age group and PGS as grouping variables, we examined the effects of age and PGS on learning and pre-test performance by task type. Age group (1 = older, 2 = younger) and PGS

were centered at 0 (standardized) to interpret the main effect. Refer to Supplementary Tables 7-9 and Supplementary Tables 10-12 for parameter estimates of EAPGS and CPPGS models, respectively. Age displayed significant correlations with pre-test performance, where younger age group was associated with higher pre-test scores for all task types. Age also showed significant correlations with the difference in performance between pre- and post-test for all cognitive tasks. For episodic memory and working memory tasks, younger age group was associated with smaller differences between pre- and post-test performance compared to the older group. This could be explained by ceiling effect, where younger adults already showed high performance at pre-test and the high scorers were in a very difficult starting position, as they may have already been too close to their upper limit for improvement. In contrast, younger age group was associated with larger differences in performance between pre- and post-test for processing speed tasks. This finding is in line with many studies that have found that cognitive processing slows with age[3–5].

CPPGS displayed positive correlation with pre-test scores of episodic memory tasks. This suggests that individuals with higher CPPGS are more likely to score higher than those with lower CPPGS. No main effects of PGS were found in any other task types. In addition, the interactions between age and PGS were not significant. This indicates that for most of the time, the effect of PGS on change did not differ reliably as a function of age group. In general, the results from these analyses were in line with those from the regression analyses, in that they showed small effects.

d. Performance as correlates of pre-test scores by age group. We correlated pre-test scores with performance for older and younger groups to examine age differences in the shape of learning curves (Supplementary Figure 6). We observe higher levels of correlations among younger groups in five of the nine cognitive tasks (Word List Memory Task, Number-Noun Pairs, Object Position Memory, Memory Updating Numerical, and Figural/Spatial Comparison), suggesting that participants in the younger age group show more differences in performance between participants with high and low scores at baseline than those in the older age group. In particular, the gaps between older and younger adults are most prominent in Figural/Spatial Comparison. However, all cognitive tasks show overlaps in correlations between older and younger groups, indicating little to no effect of age. Figural/Spatial Comparison was the only cognitive task that showed distinguished differences in correlations between the two age groups that begins towards the latter half of the training phase.

e. Performance as correlates of EAPGS by age group. We identified age differences in the shape of learning curves by separately correlating EAPGS with performance for older and younger age groups (Supplementary Figure 8). In all cognitive tasks except for Alpha Span, we observe substantial similarities in trajectories of correlation, indicating that the influence of EAPGS on the shape of learning is consistent over age. In Alpha Span, there is an upward trend in correlations from negative correlations for both age groups with slightly more visible increase in the level of correlations in the older group. This suggests that older participants with lower EAPGS perform better at baseline, but the difference between the high and low EAPGS equalize towards the end of the training phase. The correlations are close to zero and there is minimal change among younger participants, indicating similar rates of learning across the entire training phase.

f. Performance as correlates of CPPGS. We also examined the correlations of CPPGS with performance for all cognitive tasks. Like previous analyses, CPPGS and cognitive scores at all individual waves were computed to be independent of age at pre-test and PC of ancestry prior to analyses. Correlations between CPPGS and cognitive task performance ranged from approximately -.2 to .25, indicating little to no clear differences between participants between high and low PGS scores.

Two types of correlation trajectories were identified in correlations between CPPGS and performance. All three tasks that measured perceptual speed and two of the three tasks that measured working memory (Memory Updating Numerical and N-Back Spatial) showed a downward trend in correlations that result in negative correlations, suggesting that participants with high CPPGS start the practice sessions off with slight advantage, but the difference between high and low PGS groups equalize over time and even lead to the low PGS group performing better (see Supplementary Figure 9). On the other hand, all three tasks that measured episodic memory plus Alpha Span show correlations that generally remain constant throughout the longitudinal training period. With correlations in the close to 0, this trend suggests that CPPGS has no strong effect on learning. The correlations and their trajectories remain similar, even when creating means for blocks of 10 sessions. See Supplementary Figure 10 for correlations between CPPGS and the average performance across blocks of 10 days. We also examined performance as correlates of CPPGS by age group (Supplementary Figure 11). Like previous analyses that examine age differences in performance as correlates, most cognitive tasks show overlaps in correlations between older and younger groups, indicating little age differences. However, the gaps between older and younger adults in all cognitive tasks except for Alpha Span and Numerical show to be more pronounced than those for correlations between EAPGS and cognitive task performance (see Supplementary Figures 8 and 11). In particular, the gaps between the two age groups in Word List Memory Task and Verbal are larger for correlations between CPPGS and cognitive task performance, where there is little or no overlaps in correlations between the two groups in comparison to the age group gaps for correlations between EAPGS and performance.

2. Supplementary Figure Legends

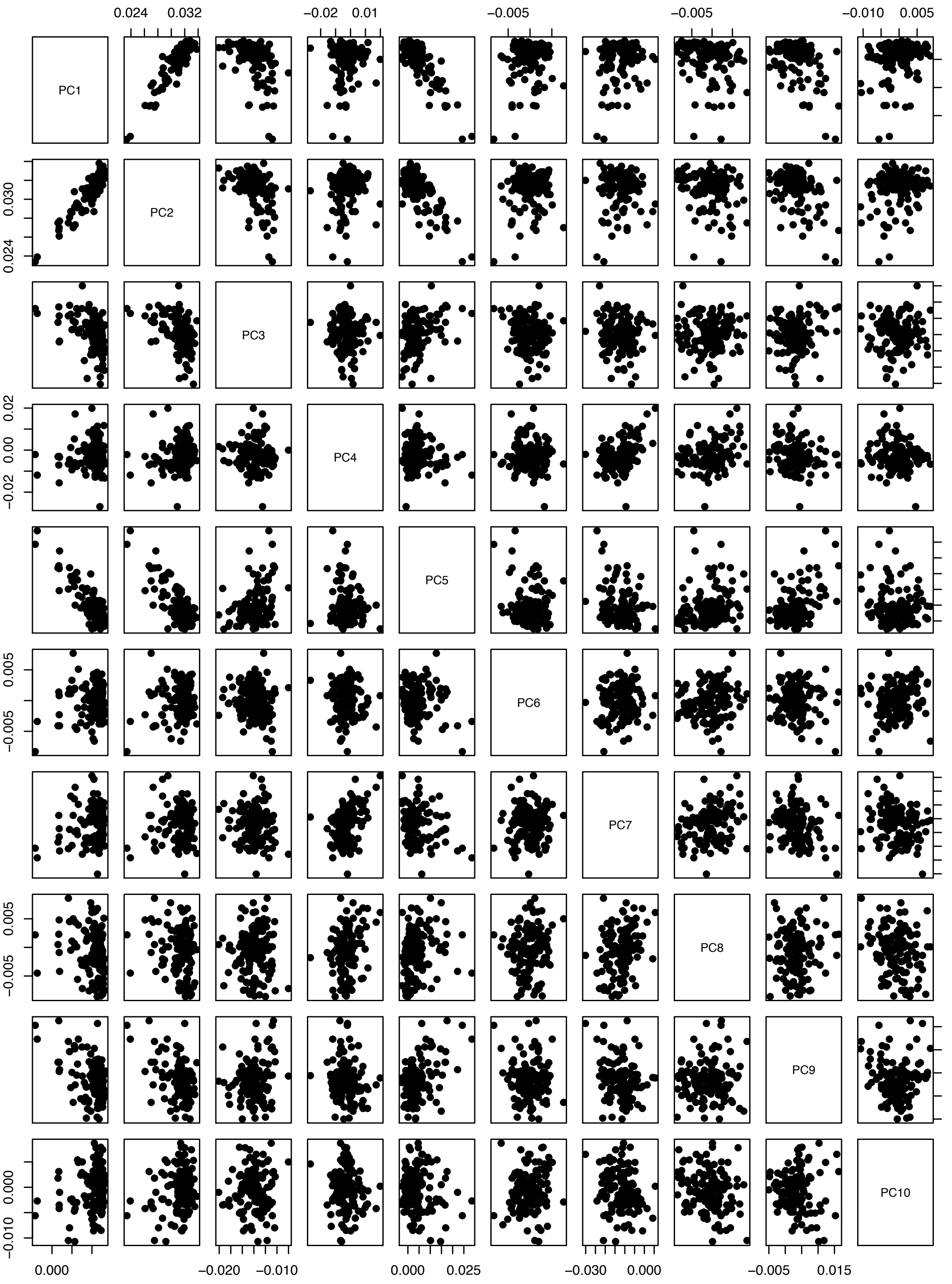

Supplementary Figure 1. **Scatterplot matrix of the 10 principal components (PC).**

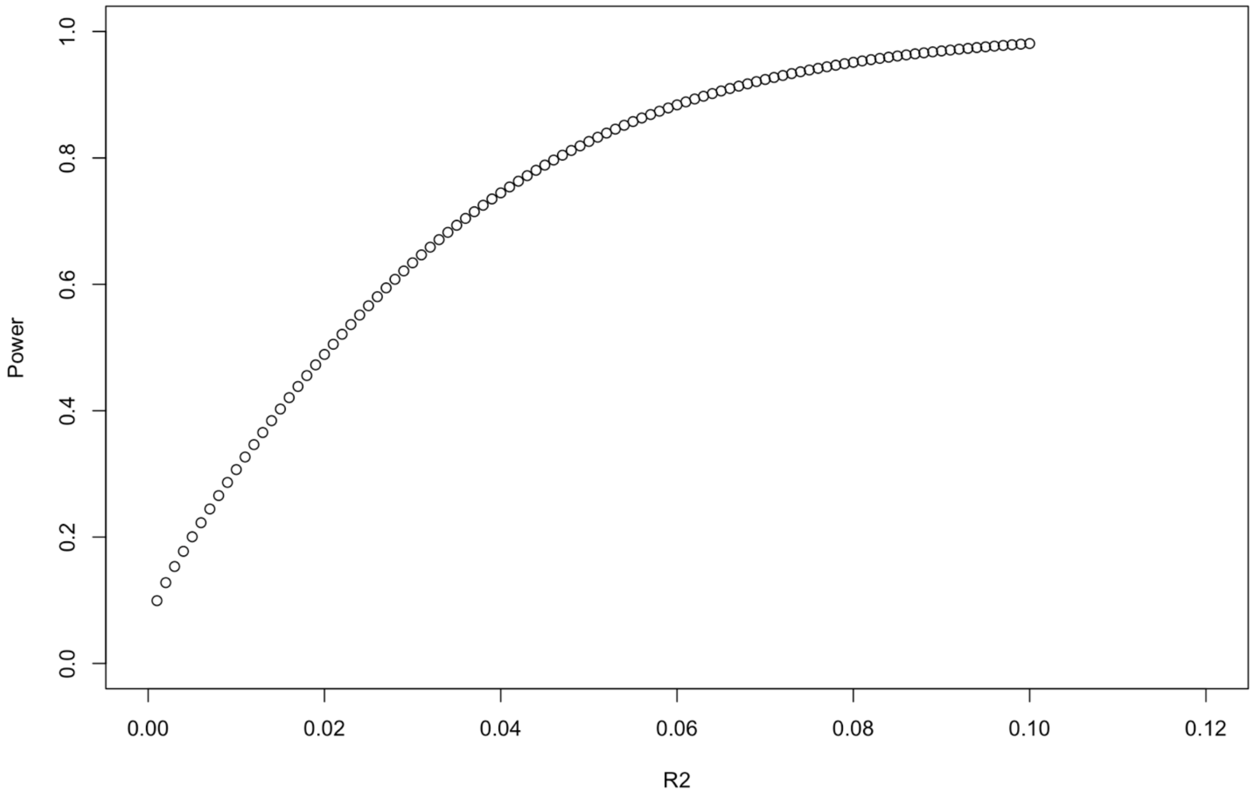

Supplementary Figure 2. **Estimated power of the model with respect to the predictive power of PGS.**

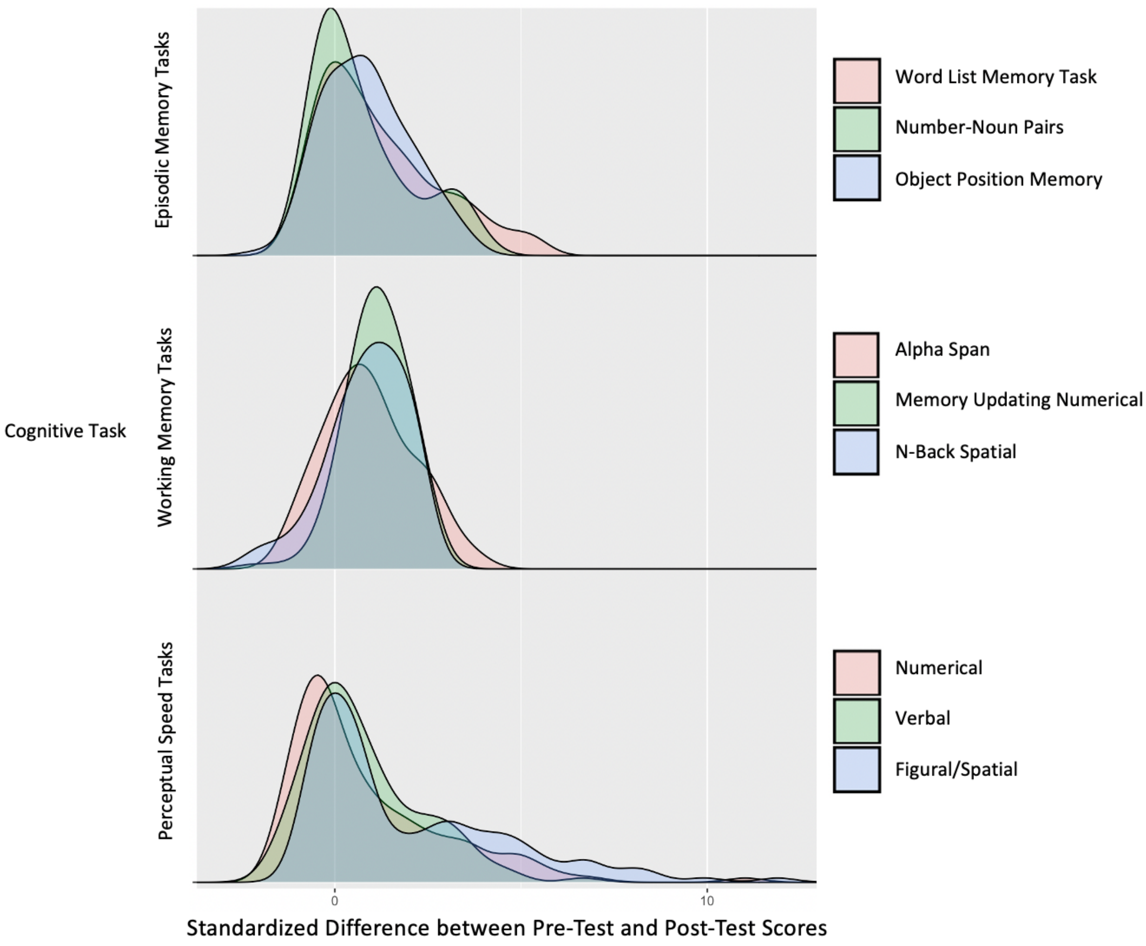

***Supplementary Figure 3.* Ridge plot of the distribution of the standardized differences between pre-test and post-test scores by task type.**

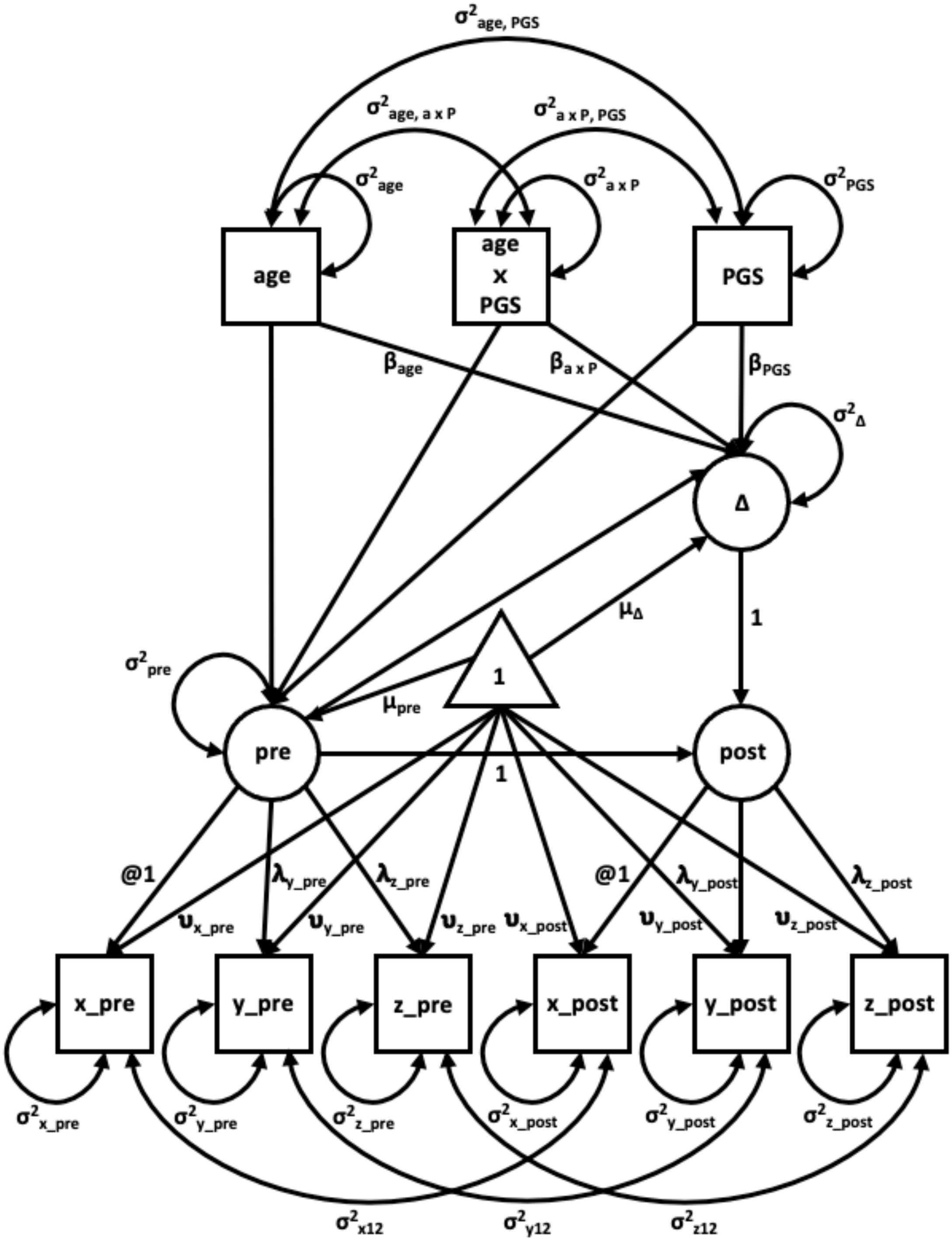

Supplementary Figure 4. **Path diagram of the LDSM used in the analyses.**
Squares represent observed variables, and circles represent latent variables, including baseline (pre) and follow-up (post) occasions and a latent difference variable (Δ). The triangle represents estimated means. Single-headed arrows represent regressions, and double-headed arrows represent variance and covariance relations.

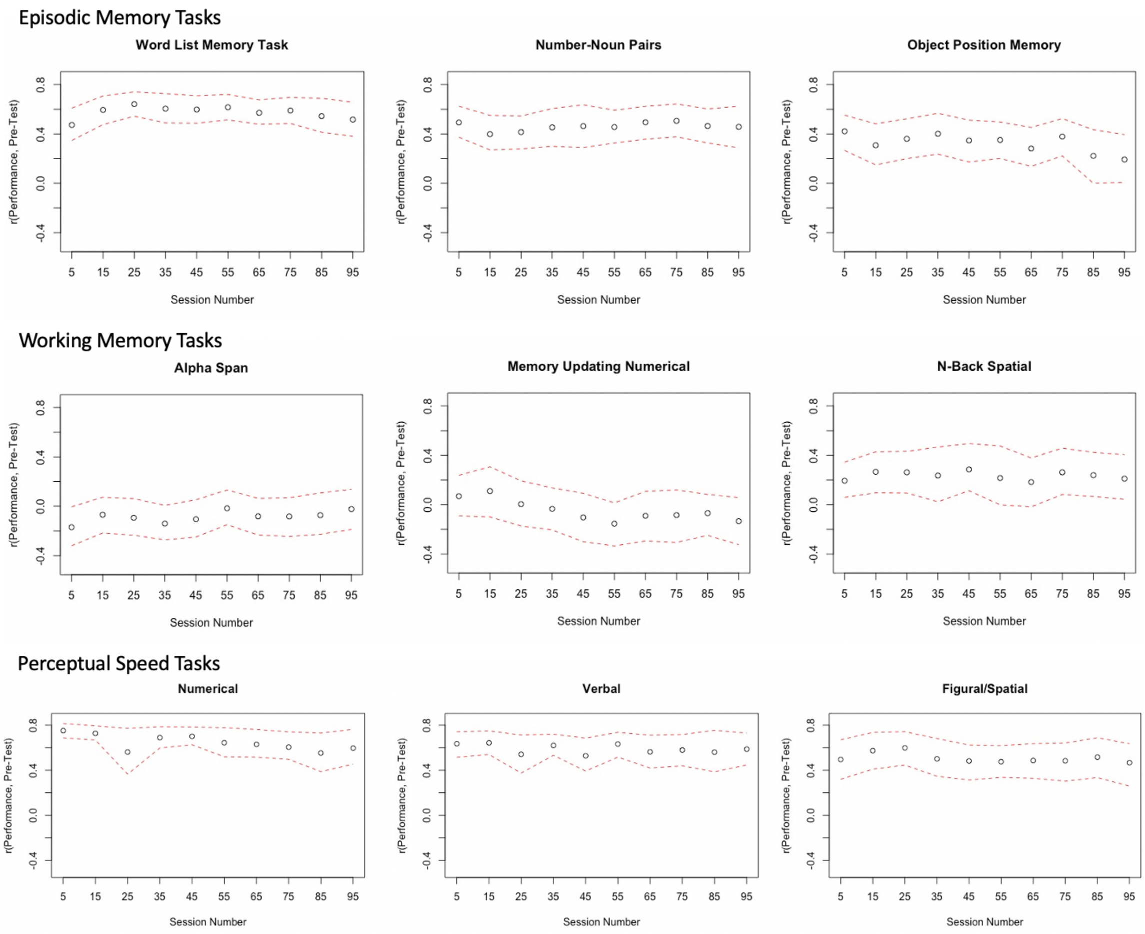

***Supplementary Figure 5*. Paneled plot of correlations between pre-test scores and performance on all cognitive tasks over the training phase across blocks of 10 days.** The black circles indicate correlations between pre-test scores and performance, and the red dotted lines indicate 95% confidence intervals.

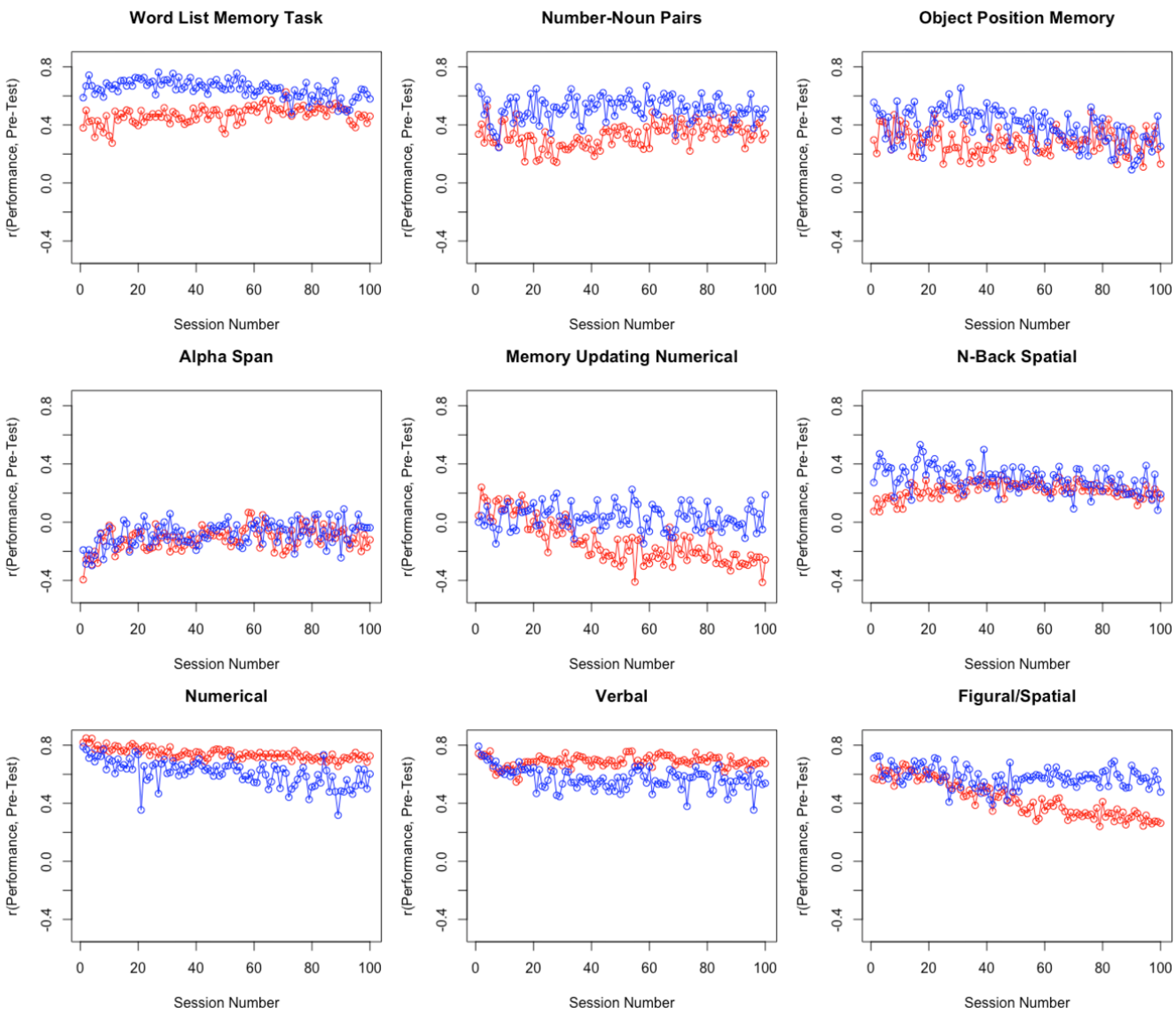

***Supplementary Figure 6*. Paneled plot of correlations between pre-test scores and performance on all cognitive tasks over the training phase by age group.**

The red and blue lines indicate correlations between pre-test scores and performance for older and younger age group, respectively.

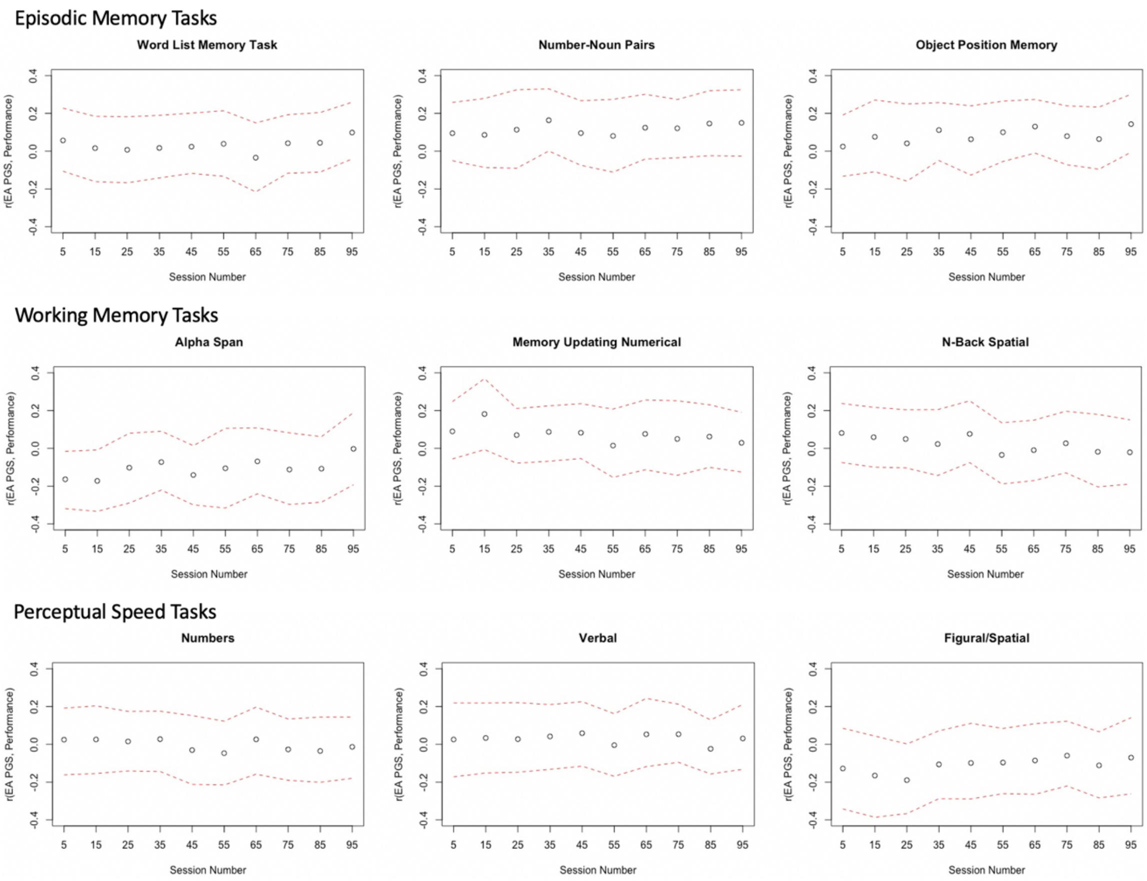

***Supplementary Figure 7*. Paneled plot of correlations between EAPGS and performance on all cognitive tasks over the training phase across blocks of 10 days.**
The black circles indicate correlations between EAPGS and performance, and the red dotted lines indicate 95% confidence intervals.

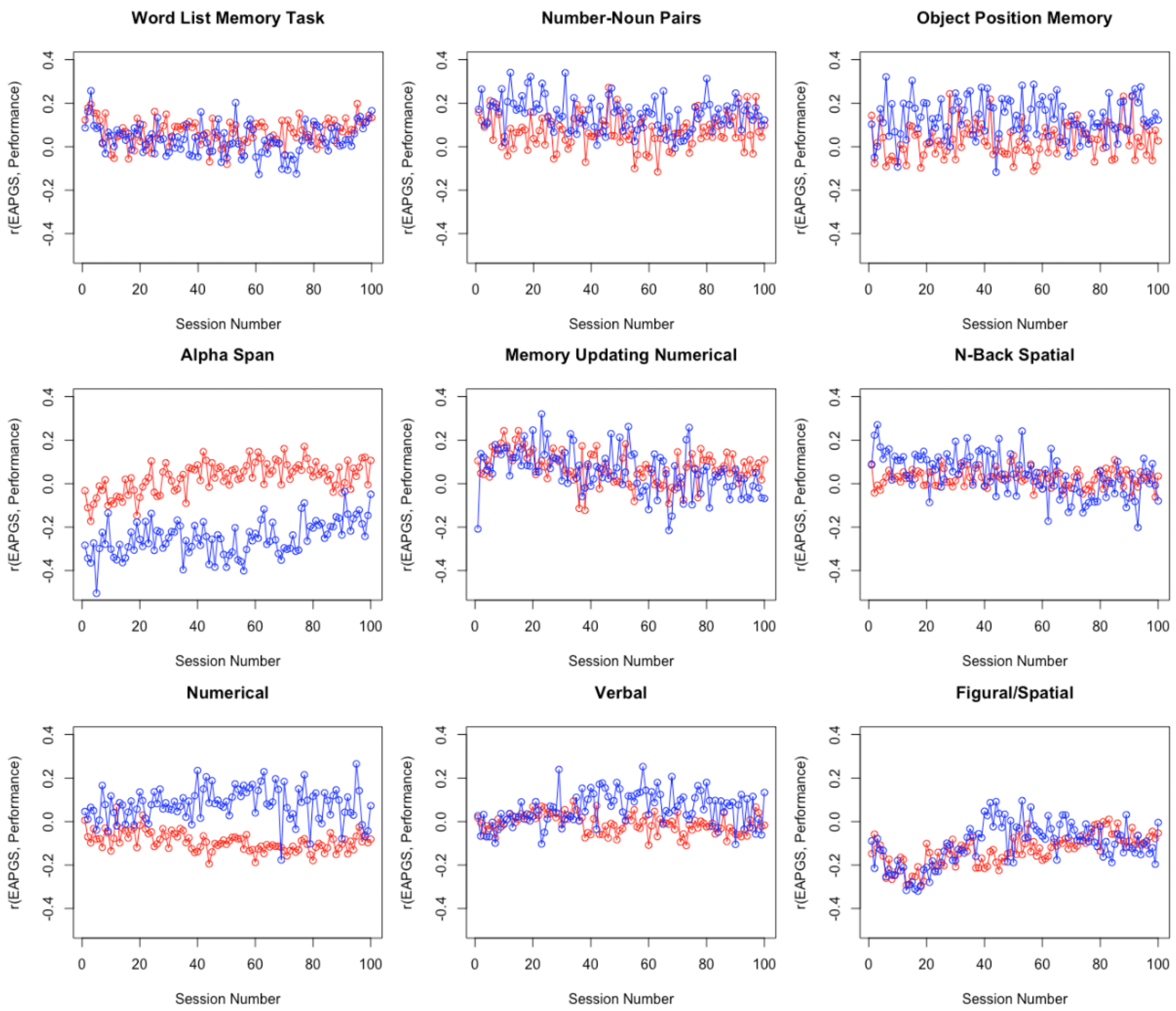

***Supplementary Figure 8*. Paneled plot of correlations between EAPGS and performance on all cognitive tasks over the training phase by age group.**

The red and blue lines indicate correlations between pre-test scores and performance for older and younger age group, respectively.

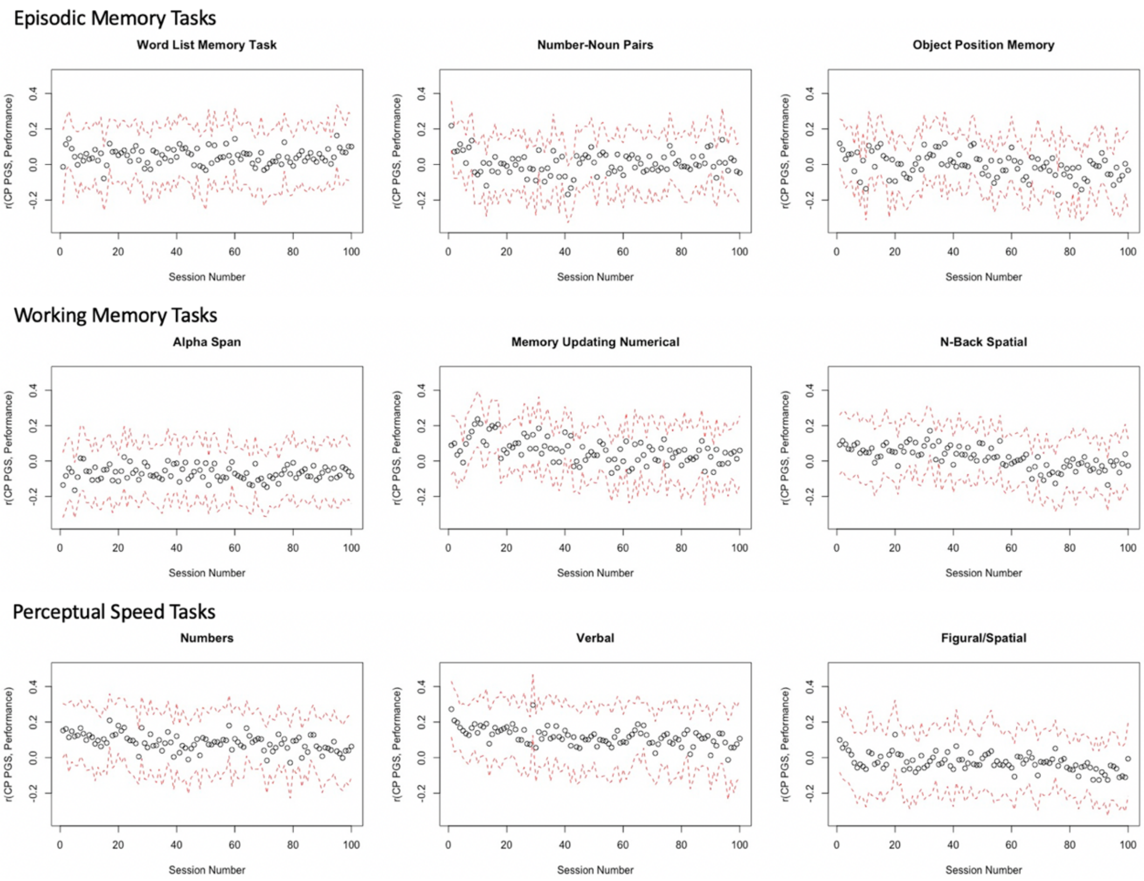

***Supplementary Figure 9*. Paneled plot of correlations between CPPGS and performance on all cognitive tasks over the training phase.**
The black circles indicate correlations between CPPGS and performance, and the red dotted lines indicate 95% confidence intervals.

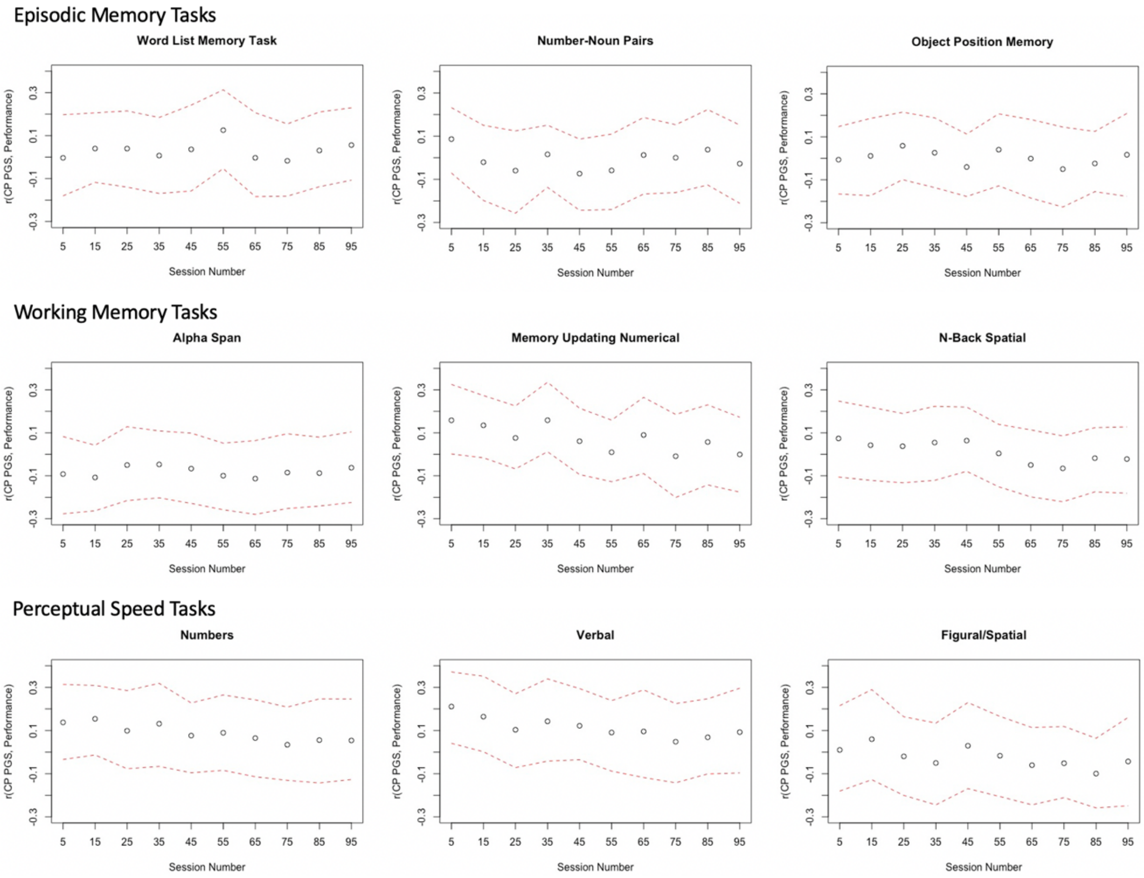

Supplementary Figure 10. **Paneled plot of correlations between CPPGS and performance on all cognitive tasks over the training phase across blocks of 10 days.**

The black circles indicate correlations between CPPGS and performance, and the red dotted lines indicate 95% confidence intervals.

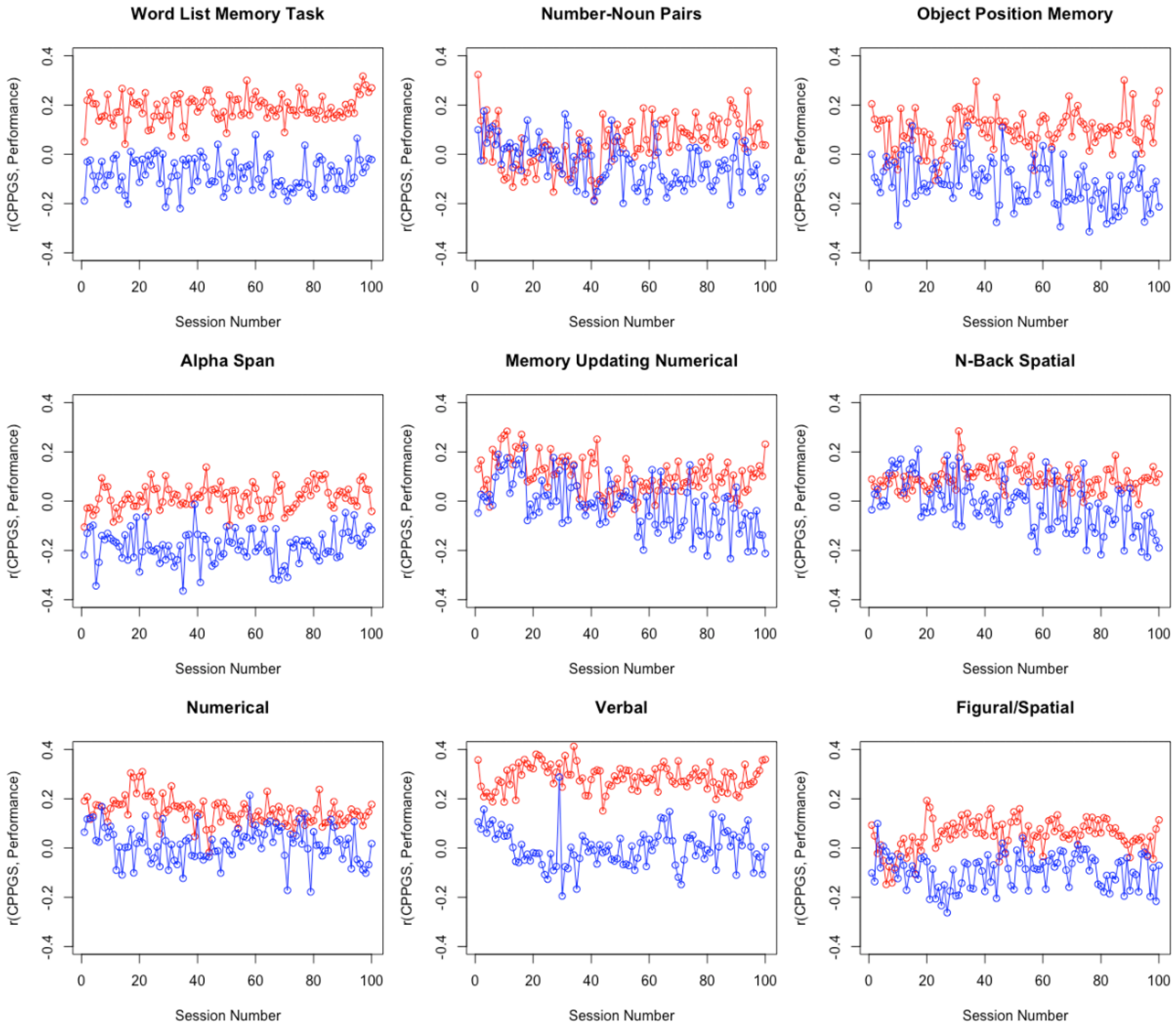

***Supplementary Figure 11*. Paneled plot of correlations between CPPGS and performance on all cognitive tasks over the training phase by age group.**

The red and blue lines indicate correlations between pre-test scores and performance for older and younger age group, respectively.

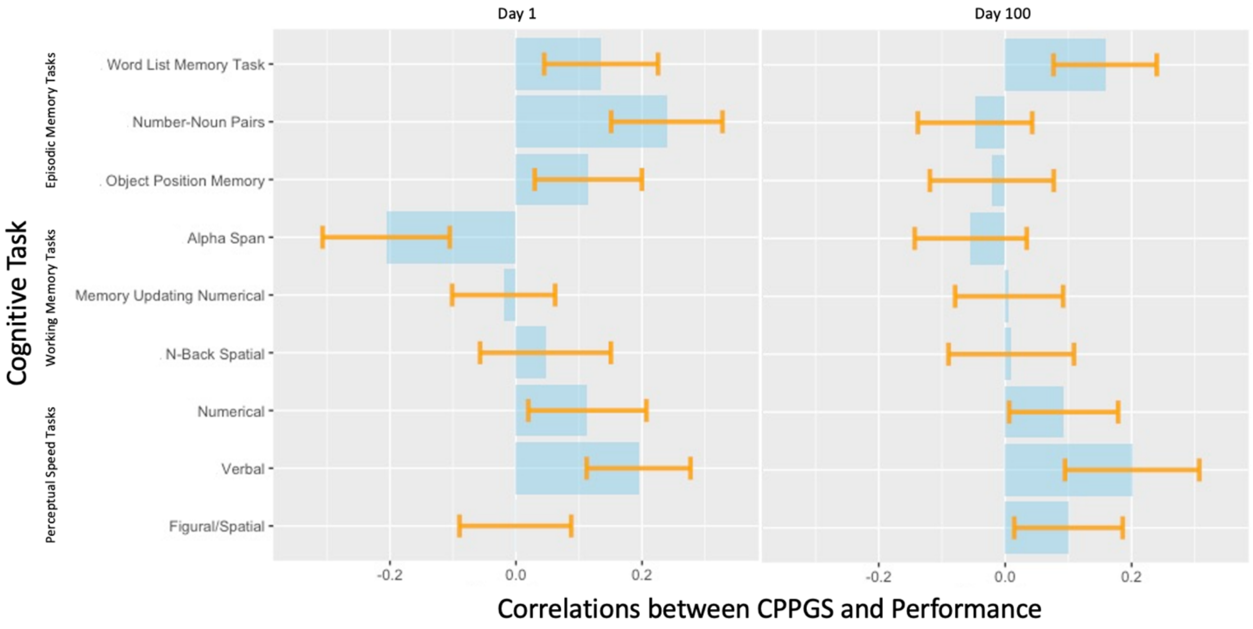

***Supplementary Figure 12*. Spearman correlations between CPPGS and performance at day 1 (top) and at day 100 of the training period (bottom) on all cognitive tasks.**
The blue bars indicate correlation coefficients between CPPGS and test performance and orange lines indicate error bars (i.e., ± standard error). Spearman correlations were used to reduce the potential influence of outliers.

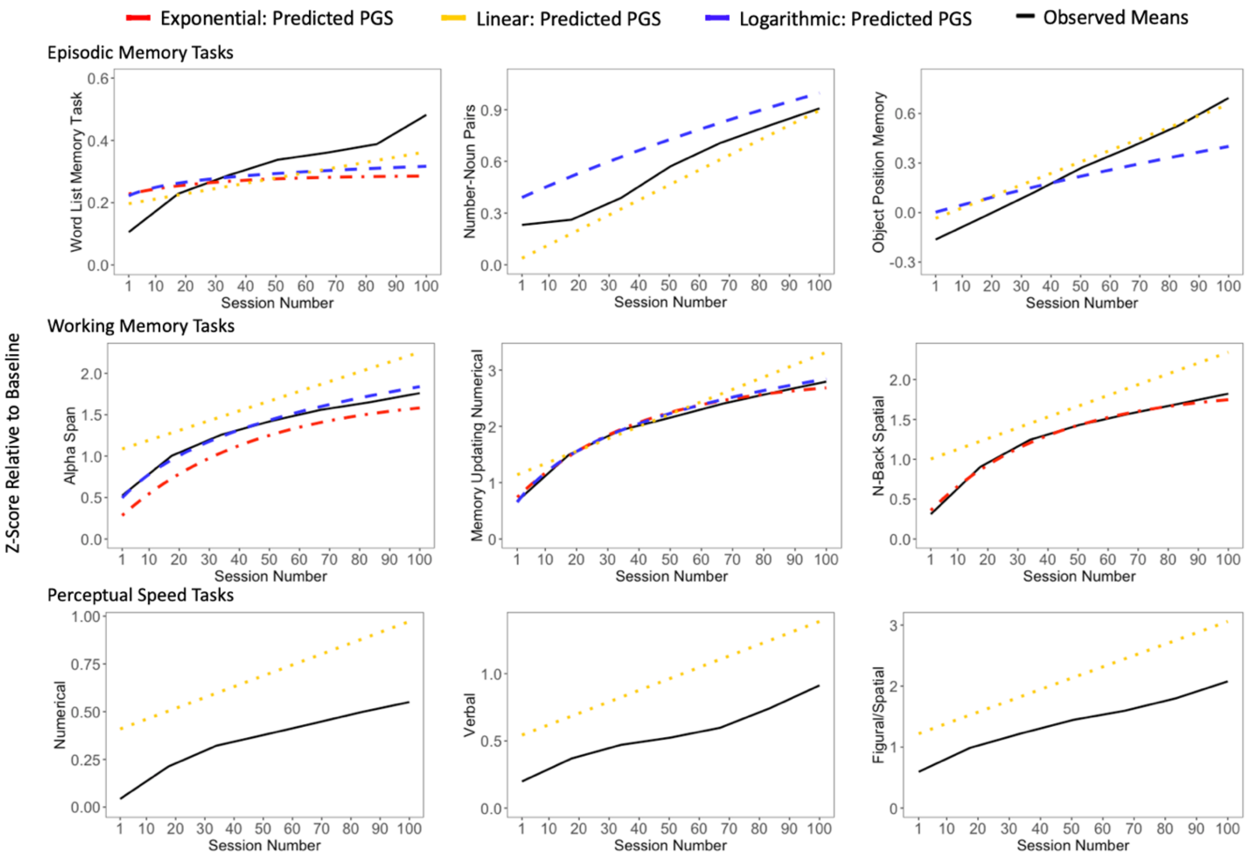

***Supplementary Figure 13*. Paneled plot of means in performance over the training phase for all participants on all cognitive tasks**

3. Supplementary Tables

***Supplementary Table 1*. Descriptions of Cognitive Tasks**

Cognitive Task	Specific Domain	Description
Word List Memory Task	Visual episodic memory	Lists of 36 nouns were demonstrated in sequence with PT of 1000, 2000, or 4000 ms, depending on the pre-test performance of the participant. The ISI was 1000 ms. Frequencies and lengths of word, emotional valence, and imaginability were equally balanced across word lists. After a list was presented, participants were instructed to enter the first three letters of each word in a correct order. Two trials were conducted per daily session. The performance score was measured by multiplying the number of correctly recalled words by the score of word order accuracy, ranging from 0 to 1. The final scores were logit transformed for statistical analyses.
Number-Noun Pairs	Numerical episodic memory	Lists of 12 two-digit numbers and plural nouns (e.g., "22 dogs") were presented sequentially with the individually adjusted PT of 1000, 2000, or 4000 ms and the ISI of 1000 ms. After each presentation, participants were instructed to enter the corresponding numbers of randomly ordered nouns. Two trials were conducted per daily session. The percentage of correctly recalled numbers were logit transformed.
Object Position Memory	Figural-spatial episodic memory	Series of 12 colored photographs of real-world objects were presented at different positions in a 6x6 grid with the individually adjusted PT of 1000, 2000, or 4000 ms and the ISI of 1000 ms. After presentation, participants used the computer to move the objects back to the original places in the correct order. Two trials were conducted per daily session. The performance score was measured by multiplying the percentage of correctly placed items by the score of item order accuracy, ranging from 0 to 1. The final scores were logit transformed.
Alpha Span	Visual working memory	Ten uppercase consonants were sequentially presented with a number below each letter. For each item, participants were instructed to quickly determine if the numbers aligned with the correct alphabetical locations. Five out of the ten letters were targets with the correct position numbers. Depending on the pre-test performance of participant, the items were presented with the individually adjusted PT of 750, 1500, or 3000 ms and the ISI of 500 ms. Eight trials were conducted per daily session.
Memory Updating Numerical	Numerical working memory	Four single digits were presented simultaneously in a horizontally situated cell for 4000 ms. After an ISI of 500 ms, a series of eight updating operations ranging from -8 to +8 were displayed in the row of four cells below the previous row, requiring participants to repeatedly add or subtract random numbers and memorize the updated results. At the end of each trial, participants were instructed to enter the final answers in the four cells of the upper row, where the single digits were first

		presented. All answers ranged from 0 to 9. Depending on his or her pre-test performance, each participant was given the individually adjusted PT of 500, 1250, or 2750 ms and the ISI of 250 ms. Eight trials were conducted per daily session. The percentages of correct responses were averaged across odd and even blocks and logit transformed.
N-Back Spatial	Spatial working memory	A series of black dots were presented in various places in a 4x4 grid with the PT of 500 ms and the individually adjusted ISI of 500, 1500, or 2500 ms, where participants were instructed to compare the positions of the dots between current and previous presentations. There were 12 targets out of a total of 39 dots. No dots were presented in the same location twice in a row, but four groups of three items presented in the same locations as the items in two, four, five, or six previous steps. Four trials were conducted per daily session. The percentages of correct responses on Trials 4 through 39 were averaged across odd and even blocks and logit transformed.
Comparison tasks	Perceptual speed	For all three versions of the comparison tasks that measure perceptual speed, participants were instructed to quickly compare two rows of numbers, letters, or figures and decide whether they were same or different. If different, only one of the numbers, letters, or figures were different. In the numerical version of the task, the two strings consisted of five single-digits. The verbal version was equivalent to that of the numerical version, with consonants instead of digits. Similarly, two two-toned three-dimensional objects ("fribbles") were placed side by side for comparison in the figural/spatial version. Each of the comparison tasks held two trials that consists of 40 items. The performance scores were measured by dividing the number of correct responses by the total response time (in seconds) and multiplying the quotient by 60 to create a score of correct responses per minute. Scores above 100 (top 0.5%) were removed to reduce the influence of outliers.

Note: PT = presentation time, ISI = interstimulus interval, ms = milliseconds

Supplementary Table 2. **Growth model equations**

Regression Model	Equation	Description of Parameters
Linear	$y_i = b_0 + b_1 * x_i + u_i$	i = individual<br>b_0 = intercept<br>b_1 = association between test score and EA/CPPGS<br>u = residuals
Latent Difference Score	$\Delta_i = b_0 + b_1 * a_i + b_2 * PGS_i + b_3 * a_i * PGS_i + b_4 * pre_i + u_i$<br>$\Delta_i = post_i - pre_i$	i = individual<br>b_0 = intercept<br>b_1 = coefficient of age on Δ<br>a = age<br>b_2 = coefficient of EA/CPPGS on Δ<br>PGS = EA/CPPGS<br>b_3 = coefficient of the interaction between age and EA/CPPGS on Δ<br>b_4 = coefficient of pre-test score on Δ<br>pre = pre-test score<br>u = residuals<br>post = post-test score
Multilevel Linear	$y_{i,t} = b_{0i} + b_{1i} * t + u_{i,t}$	i = individual<br>t = time<br>b_0 = intercept<br>b_1 = time scaling factor<br>u = residuals
Logarithmic	$y_{i,t} = b_{0i} + b_{1i} * \log(t - b_{2i}) + u_{i,t}$	i = individual<br>t = time<br>b_0 = intercept<br>b_1 = time scaling factor<br>b_2 = horizontal displacement

		u = residuals
Exponential	$y_{i,t} = b_{0i} - b_{1i}e^{b_2(t_i)} + u_{i,t}$	i = individual<br>t = time<br>b_0 = horizontal asymptote<br>b_1 = time scaling factor<br>b_2 = growth rate<br>u = residuals

***Supplementary Table 3*. Difference between pre-test scores and post-test scores**

	Standardized Post-Test Relative to Pre-Test			
	M (SD)	*SE*	95% CI	
			Lower	Upper
Episodic Memory Tasks				
Word List Memory Task	1.1613 (1.5913)	.139	.8863	1.4364
Number-Noun Pairs	.7268 (1.3174)	.1151	.4990	.9545
Object Position Memory	.8478 (1.1486)	.1004	.6493	1.046
Working Memory Tasks				
Alpha Span	.9124 (1.1861)	.1036	.7074	1.117
Memory Updating Numerical	1.1383 (.8061)	.0704	.9990	1.2777
N-Back Spatial	.8671 (1.0507)	.0918	.6855	1.049
Perceptual Speed Tasks				
Numerical Comparison	1.0139 (3.2545)	.2844	.4513	1.5764
Verbal Comparison	.8623 (2.0956)	.1831	.5001	1.2246
Figural/Spatial Comparison	2.0237 (2.8455)	.2486	1.5318	2.5155

Note: M = mean, SD = standard deviation, SE = standard error, CI = confidence interval

***Supplementary Table 4*. Linear regression analysis between CPPGS and test scores by cognitive task**

	Pre-Test Scores		Post-Test Scores		Difference	
	Estimate	*SE*	*Estimate*	*SE*	*Estimate*	*SE*
Episodic Memory Tasks						
Word List Memory Task	.0152	.0953	.0652	.0618	.1653*	.0932
Number-Noun Pairs	-.0036	.0923	.0000	.0694	.0068	.1245
Object Position Memory	-.029	-.0945	.0094	.0816	.0541	.1077
Working Memory Tasks						
Alpha Span	-.0132	.1038	-.0183	.0873	-.0265	.1502
Memory Updating Numerical	.1154	.1075	.0609	.1165	-.1083	.1411
N-Back Spatial	.0343	.0931	-.0183	.088	-.0822	.114
Perceptual Speed Tasks						
Numerical Comparison	-.0064	.088	-.0016	.0271	.0025	.0289
Verbal Comparison	.0431	.088	-.0132	.042	-.0424	.0569
Figural/Spatial Comparison	-.1095	.0875	-.0381	.0308	-.0394	.0391

Note: CPPGS = cognitive performance polygenic score; SE = standard error. * Regression is significant at the .05 level (1-tailed).

***Supplementary Table 5*. Linear regression analysis between educational attainment and test scores by cognitive task**

	Pre-Test Scores		Post-Test Scores		Difference	
	Estimate	*SE*	*Estimate*	*SE*	*Estimate*	*SE*
Episodic Memory Tasks						
Word List Memory Task	.3224***	.0910	.2196***	.0590	.1907*	.0928
Number-Noun Pairs	.3193***	.0879	.2480***	.0658	.2179*	.1230
Object Position Memory	.1946*	.0929	.1504*	.0805	.0095	.1078
Working Memory Tasks						
Alpha Span	.4259***	.0968	.3553***	.0815	.1590	.1496
Memory Updating Numerical	.4390***	.1008	.4822***	.1086	-.0438	.1414
N-Back Spatial	.4232***	.0853	.3705***	.0817	-.0125	.1141
Perceptual Speed Tasks						
Numerical Comparison	.0330***	.0831	.0338	.0269	.0030	.0289
Verbal Comparison	.3127***	.0836	.1351***	.0403	.1176*	.0560
Figural/Spatial Comparison	.0166*	.0868	.0878**	.0300	.1081**	.0380

Note: SE = standard error. * Regression is significant at the .05 level (1-tailed). ** Regression is significant at the .01 level (1-tailed). *** Regression is significant at the .001 level (1-tailed).

***Supplementary Table 6*. RMSEA and CFI for the LDSM for each PGS and task type**

	EAPGS			CPPGS		
	Episodic Memory	Working Memory	Processing Speed	Episodic Memory	Working Memory	Processing Speed
RMSEA	0.033	0.000	0.094	0.053	0.000	0.096
CFI	0.985	1.000	0.906	0.961	1.000	0.902

Note: RMSEA = root mean square error of approximation, CFI = confirmatory fit index, LDSM = latent difference score models, PGS = polygenic score, EAPGS = educational attainment polygenic score, CPPGS = cognitive performance polygenic score.

***Supplementary Table 7*. Parameter estimates from standardized age × EAPGS latent difference score models for episodic memory tasks**

Model Results	*β*	SE	*t*	R^2
Loadings				
Word List Memory Task	1.000	.000	-	
Number-Noun Pairs	1.135	.058	19.676***	
Object Position Memory	.793	.055	14.379***	
Delta ON (Constant)				
Age	-.023	.012	-1.97*	
EAPGS	.005	.008	.626	
Age × EAPGS	.001	.008	.124	
F_pre ON (Constant)				
Age	.071	.009	8.172***	
EAPGS	.009	.009	.996	
Age × EAPGS	-.004	.009	-.458	
Intercepts				
Word List Memory Task	.24	.034	7.08***	
Number-Noun Pairs	.236	.039	6.077***	
Object Position Memory	.356	.028	12.716***	
F_pre	.000	.000	-	
F_post	.000	.000	-	
Delta	.127	.032	3.951***	
Residual Variances				
Pre Word List Memory Task	.003	.001	4.225***	.835
Pre Number-Noun Pairs	.009	.001	6.752***	.658
Pre Object Position Memory	.014	.002	7.675***	.376
Post Word List Memory Task	.009	.002	5.52***	.764
Post Number-Noun Pairs	.01	.002	5.622***	.783
Post Object Position Memory	.015	.002	7.17***	.554
F_pre	.008	.001	6.386***	.405
F_post	.000	.000	-	1.000
Delta	.001	.001	.389	.98

Note: EAPGS = educational attainment polygenic score, SE = standard error, ON = regressed on, F_pre = pre-test factor model, F_post = post-test factor model
*** $p < .001$, ** $p < .01$, * $p < .05$.

***Supplementary Table 8.* Parameter estimates from standardized age × EAPGS latent difference score models for working memory tasks**

Model Results	*β*	SE	*t*	R^2
Loadings				
Alpha Span	1.000	.000	-	
Memory Updating Spatial	1.254	.081	15.412***	
N-Back Spatial	1.146	.061	18.854***	
Delta ON (Constant)				
Age	-.022	.011	-1.999*	
EAPGS	-.005	.005	-.92	
Age × EAPGS	.005	.005	1.129	
F_pre ON (Constant)				
Age	.064	.005	11.753***	
EAPGS	.01	.005	1.906	
Age × EAPGS	-.004	.005	-.829	
Intercepts				
Alpha Span	.561	.021	27.237***	
Memory Updating Spatial	.592	.027	22.005***	
N-Back Spatial	.712	.024	29.905***	
F_pre	.000	.000	-	
F_post	.000	.000	-	
Delta	.096	.016	.000	
Residual Variances				
Pre Alpha Span	.002	.000	5.937***	.741
Pre Memory Updating Spatial	.013	.002	7.177***	.433
Pre N-Back Spatial	.005	.001	7.091***	.635
Post Alpha Span	.003	.000	5.633***	.758
Post Memory Updating Spatial	.006	.001	6.603***	.656
Post N-Back Spatial	.005	.001	6.786***	.657
F_pre	.002	.000	5.042***	.645
F_post	.000	.000	-	1.000
Delta	-.001	.000	-1.269	-

Note: EAPGS = educational attainment polygenic score, SE = standard error, ON = regressed on, F_pre = pre-test factor model, F_post = post-test factor model
*** p < .001, ** p < .01, * p < .05.

***Supplementary Table 9.* Parameter estimates from standardized age × EAPGS latent difference score models for processing speed tasks**

Model Results	*β*	SE	*t*	R^2
Loadings				
Numerical	1.000	.000	-	
Verbal	.772	.041	18.727***	
Figural/Spatial	.706	.045	15.538***	
Delta ON (Constant)				
Age	.084	.02	4.117***	
EAPGS	.004	.013	.283	
Age × EAPGS	-.011	.013	-.852	
F_pre ON (Constant)				
Age	.082	.008	9.709***	
EAPGS	.011	.008	1.319	
Age × EAPGS	-.006	.008	-.749	
Intercepts				
Numerical	.62	.032	19.603***	
Verbal	.515	.024	21.157***	
Figural/Spatial	.428	.025	18.775***	
F_pre	.000	.000	-	
F_post	.000	.000	-	
Delta	.062	.051	1.225	
Residual Variances				
Pre Numerical	.004	.001	6.006***	.776
Pre Verbal	.001	.000	3.506***	.865
Pre Figural/Spatial	.004	.001	6.677***	.614
Post Numerical	.078	.009	8.783***	.537
Post Verbal	-.009	.002	-5.03***	-
Post Figural/Spatial	.039	.005	7.519***	.537
F_pre	.007	.001	6.214***	.494
F_post	.000	.000	-	1.000
Delta	.044	.007	6.797***	.511

Note: EAPGS = educational attainment polygenic score, SE = standard error, ON = regressed on, F_pre = pre-test factor model, F_post = post-test factor model
*** p < .001, ** p < .01, * p < .05.

***Supplementary Table 10.* Parameter estimates from standardized age × CPPGS latent difference score models for episodic memory tasks**

Model Results	β	SE	t	R^2
Loadings				
Word List Memory Task	1.000	.000	-	
Number-Noun Pairs	1.145	.058	19.675***	
Object Position Memory	.798	.056	14.321***	
Delta ON (Constant)				
Age	-.027	.012	-2.233*	
CPPGS	-.007	.009	-.783	
Age × CPPGS	-.005	.008	-.586	
F_pre ON (Constant)				
Age	.071	.009	8.296***	
CPPGS	.018	.009	2.059*	
Age × CPPGS	.008	.008	.922	
Intercepts				
Word List Memory Task	.238	.033	7.156***	
Number-Noun Pairs	.234	.038	6.074***	
Object Position Memory	.355	.028	12.792***	
F_pre	.000	.000	-	
F_post	.000	.000	-	
Delta	.125	.032	3.9***	
Residual Variances				
Pre Word List Memory Task	.003	.001	4.475***	.822
Pre Number-Noun Pairs	.009	.001	6.682***	.667
Pre Object Position Memory	.014	.002	7.681***	.378
Post Word List Memory Task	.009	.002	5.581***	.757
Post Number-Noun Pairs	.01	.002	5.503***	.786
Post Object Position Memory	.015	.002	7.14***	.555
F_pre	.007	.001	6.225***	.431
F_post	.000	.000	-	1.000
Delta	.000	.001	.125	.994

Note: CPPGS = cognitive performance polygenic score, SE = standard error, ON = regressed on, F_pre = pre-test factor model, F_post = post-test factor model
*** p < .001, ** p < .01, * p < .05.

***Supplementary Table 11.* Parameter estimates from standardized age × CPPGS latent difference score models for working memory tasks**

Model Results	*β*	SE	*t*	R^2
Loadings				
Alpha Span	1.000	.000	-	
Memory Updating Spatial	1.258	.082	15.386***	
N-Back Spatial	1.155	.061	18.876***	
Delta ON (Constant)				
Age	-.023	.011	-2.09*	
CPPGS	-.005	.005	-.94	
Age × CPPGS	-.007	.005	-1.544	
F_pre ON (Constant)				
Age	.063	.005	11.634***	
CPPGS	.007	.005	1.239	
Age × CPPGS	.008	.005	1.615	
Intercepts				
Alpha Span	.559	.021	27.16***	
Memory Updating Spatial	.589	.027	21.862***	
N-Back Spatial	.71	.024	29.627***	
F_pre	.000	.000	-	
F_post	.000	.000	-	
Delta	.097	.016	6.169***	
Residual Variances				
Pre Alpha Span	.002	.000	6.05***	.729
Pre Memory Updating Spatial	.013	.002	7.214***	.434
Pre N-Back Spatial	.005	.001	7.009***	.645
Post Alpha Span	.003	.000	5.681***	.756
Post Memory Updating Spatial	.007	.001	6.625***	.654
Post N-Back Spatial	.005	.001	6.747***	.663
F_pre	.002	.000	5***	.645
F_post	.000	.000	-	1.000
Delta	-.001	.000	-1.405	-

Note: CPPGS = cognitive performance polygenic score, SE = standard error, ON = regressed on, F_pre = pre-test factor model, F_post = post-test factor model
*** p < .001, ** p < .01, * p < .05.

***Supplementary Table 12.* Parameter estimates from standardized age × CPPGS latent difference score models for processing speed tasks**

Model Results	*β*	SE	*t*	R^2
Loadings				
Numerical	1.000	.000	-	
Verbal	.768	.041	18.824***	
Figural/Spatial	.707	.045	15.604***	
Delta ON (Constant)				
Age	.083	.021	4.044***	
CPPGS	.005	.013	.378	
Age × CPPGS	.000	.012	-.01	
F_pre ON (Constant)				
Age	.081	.008	9.619***	
CPPGS	-.007	.008	-.819	
Age × CPPGS	.000	.008	-.011	
Intercepts				
Numerical	.619	.032	19.448***	
Verbal	.514	.024	21.088***	
Figural/Spatial	.427	.023	18.605***	
F_pre	.000	.000	-	
F_post	.000	.000	-	
Delta	.063	.051	1.231	
Residual Variances				
Pre Numerical	.004	.001	5.964***	.775
Pre Verbal	.001	.000	3.627***	.858
Pre Figural/Spatial	.004	.001	6.618***	.618
Post Numerical	.078	.009	8.808***	.542
Post Verbal	-.009	.002	-4.968***	-
Post Figural/Spatial	.039	.005	7.49***	.542
F_pre	.007	.001	6.174***	.488
F_post	.000	.000	-	1.000
Delta	.044	.007	6.747***	.517

Note: CPPGS = cognitive performance polygenic score, SE = standard error, ON = regressed on, F_pre = pre-test factor model, F_post = post-test factor model
*** p < .001, ** p < .01, * p < .05.

***Supplementary Table 13*. Model fit comparisons and key parameter estimates for regression models**

	Episodic Memory			Working Memory			Processing Speed		
	Word List Memory Task	Number-Noun Pairs	Object Position Memory	Alpha Span	Memory Updating Numerical	N-back Spatial	Numerical	Verbal	Figural/ Spatial
Linear									
Intercept (b_0)	-1.0945 (.1831)	-1.2300 (.1516)	-.8228 (.1196)	.2264 (.0802)	.0744 (.0878)	1.4411 (.3881)	40.3526 (1.7228)	34.3456 (1.7428)	31.0541 (1.7154)
b_0 ON PGS	.1369 (.1863)	.3356 (.1542)	.0870 (.1218)	-.0943 (.0817)	.1367 (.0893)	-.0122 (.1838)	-.5512 (.17533)	-.4348 (1.7726)	-3.6333 (1.7447)
SD of b_0	.9931 (.2689)	.6695 (.1842)	.3986 (.1130)	.1832 (.0508)	.2169 (.0618)	.9930 (.5799)	85.5108 (23.4268)	90.5432 (24.353)	87.6109 (23.5929)
Time Scaling Factor (b_1)	.0022 (.0016)	.0085 (.0017)	.0055 (.0011)	.0063 (.0009)	.0133 (.0020)	.0110 (.0037)	.0509 (.0144)	.0652 (.0163)	.1103 (.0225)
b_1 ON PGS	-.0000 (.0016)	.0002 (.0017)	-.0009 (.0011)	.0002 (.0009)	-.0001 (.0021)	.0001 (.0018)	-.0022 (.0147)	.0033 (.0166)	.0216 (.0229)
SD of b_1	.0001 (.0000)	.0001 (.0000)	.0000 (.0000)	.0000 (.0000)	.0001 (.0000)	.0001 (.0001)	.0059 (.0016)	.0078 (.0021)	.0150 (.0041)
b_0 WITH b_1	-.0001 (.0016)	.0004 (.0014)	.0008 (.0007)	-.0001 (.0004)	-.0009 (.0010)	.0044 (.0043)	.1216 (.1403)	.1862 (.1648)	.4144 (.2319)
AIC	5337.6	6545.9	5729.8	1417.4	5550.6	1329.8	15742.8	16434.0	16976.4
BIC	5343.2	6551.5	5735.3	1422.8	5556.2	1330.2	15748.3	16439.6	16982.0
Error Variance	.3201 (.0084)	.4882 (.0128)	.4010 (.0107)	.0871 (.0023)	.3470 (.0091)	.2766 (.0140)	13.6376 (.3661)	13.9309 (.3668)	16.1806 (.4248)
Logarithmic									
Intercept (b_0)	-1.1607 (.0915)	-7.3926	-4.9437 (2.6552)	-1.2376 (.14)	-1.8722 (.3293)	NC	NC	NC	NC
b_0 ON PGS	.0559 (.0649)	-.0477 (.1921)	.0584 (.0689)	-.0934 (.0564)	-.199 (.0998)	NC	NC	NC	NC
SD of b_0	3.0111 (.3711)	10.2432 (.5483)	11.2818 (.8756)	2.7366 (.3374)	3.6067 (.8282)	NC	NC	NC	NC
Time Scaling Factor (b_1)	.0481 (.0303)	1.2741	.7835 (.4931)	.3924 (.0323)	.6323 (.0728)	NC	NC	NC	NC
b_1 ON PGS	.0027 (.004)	.0512 (.0314)	.0036 (.0098)	.0126 (.0127)	.0778 (.0282)	NC	NC	NC	NC
SD of b_1	.7036 (.0733)	1.8065 (.0903)	2.0357 (.1293)	.5701 (.063)	-.847 (.1516)	NC	NC	NC	NC
Horizontal Displacement (b_2)	-7.2681 (1.2664)	-165.53	-200.16 (19.8834)	-17.9426 (2.4412)	-12.9109 (3.8954)	NC	NC	NC	NC
b_2 ON PGS	-.4209 (.5818)	10.0054 (6.6011)	1.1298 (4.9499)	-.2902 (.7436)	-1.9296 (.8833)	NC	NC	NC	NC
SD of b_2	354.82 (228.61)	-80.4317	3.2193	56.863 (8.3503)	35.7201 (11.3894)	NC	NC	NC	NC

b_0 WITH b_1	-.947 (.0127)	-.9891 (.0013)	-.9905 (.0005)	-.9773 (.0055)	.9809 (.0061)	NC	NC	NC	NC
b_0 WITH b_2	.947 (.01151)	-.8395	3.8445	.9451 (.0107)	.9473 (.0119)	NC	NC	NC	NC
b_1 WITH b_2	-.8774 (.0239)	.6646	4.7054	-.8999 (.0188)	.8945 (.0225)	NC	NC	NC	NC
AIC	19158	27009	25576	3868	19940	NC	NC	NC	NC
BIC	19195	29046	25613	3905.3	19977	NC	NC	NC	NC
Error Variance	.231 (.0029)	.4399 (.0055)	.3953 (.005)	.0709 (.0009)	.249 (.0031)	NC	NC	NC	NC
Exponential									
Horizontal Asymptote (b_0)	-.9745 (.0891)	NC	NC	.5848 (.0481)	1.1255 (.0879)	2.1645 (.1114)	NC	NC	NC
b_0 ON PGS	.1136 (.0592)	NC	NC	-.024 (.0393)	.1556 (.0645)	.1322 (.0826)	NC	NC	NC
SD of b_0	1.1453 (.0754)	NC	NC	.5968 (.0487)	1.1436 (.0879)	1.4353 (.098)	NC	NC	NC
Time Scaling Factor (b_1)	-.08 (.0729)	NC	NC	-.7992 (.0429)	-1.3131 (.0829)	-1.267 (.0015)	NC	NC	NC
b_1 ON PGS	-.0122 (.012)	NC	NC	-.0477 (.0302)	-.0633 (.0612)	.0201 (.0589)	NC	NC	NC
SD of b_1	.8494 (.0583)	NC	NC	.5356 (.0476)	1.045 (.0815)	1.1753 (.0854)	NC	NC	NC
Growth Rate (b_2)	.0352 (.0023)	NC	NC	.0221 (.0016)	.02539 (.0018)	.02466 (.0015)	NC	NC	NC
b_2 ON PGS	.008 (.0017)	NC	NC	-.0027 (.0009)	-.0039 (.0014)	-.0058 (.0009)	NC	NC	NC
SD of b_2	.676 (.622)	NC	NC	.0286 (.0026)	.0248 (.0029)	.03675 (.004)	NC	NC	NC
b_0 WITH b_1	-.8045 (.0373)	NC	NC	-.7856 (.0423)	-.9093 (.019)	-.8652 (.0247)	NC	NC	NC
b_0 WITH b_2	-.4461 (.0774)	NC	NC	-.5269 (.0879)	-.6808 (.0555)	-.5247 (.0704)	NC	NC	NC
b_1 WITH b_2	.2638 (.1027)	NC	NC	.5384 (.0907)	.6204 (.0705)	.4763 (.0794)	NC	NC	NC
AIC	19214	NC	NC	3081.9	19984	17870	NC	NC	NC
BIC	19251	NC	NC	3119.2	20021	17907	NC	NC	NC
Error Variance	.2324 (.0029)	NC	NC	.0669 (.0009)	.2496 (.0031)	.2078 (.0026)	NC	NC	NC

Note: PGS = polygenic score; SD = standard deviation; AIC = Akaike information criterion; BIC = Bayesian information criterion; NC = no convergence

4. Supplementary References

1. Muthén, L. K. & Muthén, B. O. Mplus Editor.
2. Hu, L. & Bentler, P. M. Cutoff criteria for fit indexes in covariance structure analysis: Conventional criteria versus new alternatives. *Struct. Equ. Model. A Multidiscip. J.* **6**, 1–55 (1999).
3. Cerella, J. & Hale, S. The rise and fall in information-processing rates over the life span. *Acta Psychol. (Amst).* **86**, 109–197 (1994).
4. Jenkins, L., Myerson, J., Joerding, J. A. & Hale, S. Converging evidence that visuospatial cognition is more age-sensitive than verbal cognition. *Psychol. Aging* **15**, 157–175 (2000).
5. Kerchner, G. A. *et al.* Cognitive processing speed in older adults: relationship with white matter integrity. *PLoS One* **7**, e50425–e50425 (2012).
