# Supplementary material for: Genetic associations with learning over 100 days of practice
Source: NPJ Sci Learn. 2022 May 4;7:7. doi: 10.1038/s41539-022-00121-2 (PMC9068685; doi:10.1038/s41539-022-00121-2)
Supplement: Supplementary file 2 — Reporting Summary [file 41539_2022_121_MOESM2_ESM.pdf]

nature portfolio

Corresponding author(s): Cherry Youn, Andrew Grotzinger, Christina Lill, Lars Bertram, Florian Schmiedek, Martin Lovden, Ulman Lindenberger, Michel Nivard, Kathryn Harden, Elliot Tucker-Drob

Last updated by author(s): Feb 10, 2022

Reporting Summary

Nature Portfolio wishes to improve the reproducibility of the work that we publish. This form provides structure for consistency and transparency in reporting. For further information on Nature Portfolio policies, see our Editorial Policies and the Editorial Policy Checklist.

Statistics

For all statistical analyses, confirm that the following items are present in the figure legend, table legend, main text, or Methods section.

n/a	Confirmed	
☐	☒	The exact sample size (*n*) for each experimental group/condition, given as a discrete number and unit of measurement
☐	☒	A statement on whether measurements were taken from distinct samples or whether the same sample was measured repeatedly
☐	☒	The statistical test(s) used AND whether they are one- or two-sided<br>*Only common tests should be described solely by name; describe more complex techniques in the Methods section.*
☐	☒	A description of all covariates tested
☐	☒	A description of any assumptions or corrections, such as tests of normality and adjustment for multiple comparisons
☐	☒	A full description of the statistical parameters including central tendency (e.g. means) or other basic estimates (e.g. regression coefficient) AND variation (e.g. standard deviation) or associated estimates of uncertainty (e.g. confidence intervals)
☐	☒	For null hypothesis testing, the test statistic (e.g. *F*, *t*, *r*) with confidence intervals, effect sizes, degrees of freedom and *P* value noted<br>*Give P values as exact values whenever suitable.*
☐	☒	For Bayesian analysis, information on the choice of priors and Markov chain Monte Carlo settings
☐	☒	For hierarchical and complex designs, identification of the appropriate level for tests and full reporting of outcomes
☐	☒	Estimates of effect sizes (e.g. Cohen's *d*, Pearson's *r*), indicating how they were calculated

Our web collection on statistics for biologists contains articles on many of the points above.

Software and code

Policy information about availability of computer code

Data collection	No software was used to collect the data in this study. This study used data collected by the COGITO study.
Data analysis	We used R, Mplus, and SAS to analyse the data. More specifically, we used the NLMIXED function within SAS to fit nonlinear growth curve models of changes.

For manuscripts utilizing custom algorithms or software that are central to the research but not yet described in published literature, software must be made available to editors and reviewers. We strongly encourage code deposition in a community repository (e.g. GitHub). See the Nature Portfolio guidelines for submitting code & software for further information.

Data

Policy information about availability of data

All manuscripts must include a data availability statement. This statement should provide the following information, where applicable:

- Accession codes, unique identifiers, or web links for publicly available datasets
- A description of any restrictions on data availability
- For clinical datasets or third party data, please ensure that the statement adheres to our policy

The data that support the findings of this study are available on request from the corresponding authors [F.S., M.L., & U.L.] from the COGITO study. The data are not publicly available as the collection, storage, use, and disclosure of personal data that could compromise research participant privacy or consent are strictly regulated in Germany. Data are however available from the authors upon reasonable request and with permission of the COGITO study.

Field-specific reporting

Please select the one below that is the best fit for your research. If you are not sure, read the appropriate sections before making your selection.

☐ Life sciences ☒ Behavioural & social sciences ☐ Ecological, evolutionary & environmental sciences

For a reference copy of the document with all sections, see nature.com/documents/nr-reporting-summary-flat.pdf

Behavioural & social sciences study design

All studies must disclose on these points even when the disclosure is negative.

Study description	Quantitative longitudinal
Research sample	Participants from research conducted by the COGITO study (approved by the Max Planck Institute for Human Development, Berlin) for whom genetic data were available
Sampling strategy	Convenience sample
Data collection	Participants practiced 12 different tasks using a computer. If participants were interested in hearing the results of their performed tasks, researcher explained the results after each practice. Researcher was blind to the experimental condition, as our study looked at PGS.
Timing	For all participants, there was a practice phase of approximately 100 days (M = 101 days), followed by a post-test evaluation. The average time elapsed between pre- and post-test was 197 days for the younger group, and 188 for the older group.
Data exclusions	Our analyses were restricted to participants for whom genetic data were available in order to compute the PGS. Of the participants whose genetic data were available. PGSA were computed based on GWAS summary data from the recent large-scale GWAS of educational attainment and cognitive performance. As our participants also participated in the Berlin Aging Study II (BASE-II), and due to restrictions made by the 23andMe corporation on the sharing of summary GWAS data, summary data were obtained for a version of Lee et al.'s GWAS that excluded BASE-II and 23andMe.
Non-participation	Attrition rate for participants was low (i.e., 15 out of 219 participants --including those who did not provide genetic data)
Randomization	Participants were allocated into different age groups (young adult vs old adult). We included age group and PGS as covariates for analyses.

Reporting for specific materials, systems and methods

We require information from authors about some types of materials, experimental systems and methods used in many studies. Here, indicate whether each material, system or method listed is relevant to your study. If you are not sure if a list item applies to your research, read the appropriate section before selecting a response.

Materials & experimental systems

n/a	Involved in the study
☒	☐ Antibodies
☒	☐ Eukaryotic cell lines
☒	☐ Palaeontology and archaeology
☒	☐ Animals and other organisms
☐	☒ Human research participants
☒	☐ Clinical data
☒	☐ Dual use research of concern

Methods

n/a	Involved in the study
☒	☐ ChIP-seq
☒	☐ Flow cytometry
☒	☐ MRI-based neuroimaging

Human research participants

Policy information about studies involving human research participants

Population characteristics	This study included 51 younger (age range: 20-31 years, M = 25.6, SD = 2.7) and 80 older adults (age range: 65-80 years, M = 70.4, SD = 4.0) from the COGITO study.
Recruitment	Participants were collected through advertisements and by word-of-mouth.
Ethics oversight	All research conducted by the COGITO study was approved by the Max Planck Institute for Human Development, Berlin and adhered to all ethical regulations regarding human subjects.

Note that full information on the approval of the study protocol must also be provided in the manuscript.
